# Supplementary material for: 3D-integrated multilayered physical reservoir array for learning and forecasting time-series information
Source: Nat Commun. 2024 Mar 6;15:2044. doi: 10.1038/s41467-024-46323-7 (PMC10917743; doi:10.1038/s41467-024-46323-7)
Supplement: Supplementary file 1 — Supplementary Information [file 41467_2024_46323_MOESM1_ESM.pdf]

3D-integrated Multilayered Physical Reservoir Array for Learning and Forecasting Time-series Information

Sanghyeon Choi[1,2,3], Jaeho Shin[1,4], Gwanyeong Park[1], Jung Sun Eo[1], Jingon Jang[1], J. Joshua Yang[2], Gunuk Wang[1,5,6*]*

[1]KU-KIST Graduate School of Converging Science and Technology, Korea University, 145 Anam-ro, Seongbuk-gu, Seoul 02841, Republic of Korea

[2]Department of Electrical and Computer Engineering, University of Southern California, Los Angeles, CA 90089, USA

[3]Present address: Department of Electrical and Computer Engineering, University of California, Santa Barbara, CA, 93106, USA

[4]Department of Chemistry, Rice University, 6100 Main Street, Houston, Texas 77005, USA

[5]Department of Integrative Energy Engineering, Korea University, 145 Anam-ro, Seongbuk-gu, Seoul 02841, Republic of Korea

[6]Center for Neuromorphic Engineering, Korea Institute of Science and Technology, Seoul 02792, Republic of Korea

**Corresponding author. E-mail:* jjoshuay@usc.edu, gunukwang@korea.ac.kr

Contents

Supplementary Figures

Supplementary Fig. 1. Schematics of the single and wide reservoir computing systems
Supplementary Fig. 2. A 16 × 16 WO_x memristive crossbar array via photolithography
Supplementary Fig. 3. Fabrication procedure of the 3D stacked crossbar arrays
Supplementary Fig. 4. A schematic of conventional recurrent neural networks
Supplementary Fig. 5. Schematics of horizontally and vertically stacked 3D arrays
Supplementary Fig. 6. All switching curves at the first layer in the 3D stacked arrays
Supplementary Fig. 7. All switching curves at the second layer in the 3D stacked arrays
Supplementary Fig. 8. All switching curves at the third layer in the 3D stacked arrays
Supplementary Fig. 9. Statistical histograms of switching parameters in the 3D stacked arrays
Supplementary Fig. 10. Cycle-to-cycle variation during the consecutive 4,000 sweeps
Supplementary Fig. 11. The lowest operating voltage range of ~0.7 V
Supplementary Fig. 12. A current–time plot for the robust self-rectification
Supplementary Fig. 13. Plots of current behaviors over time under air and vacuum condition
Supplementary Fig. 14. A plot of characteristic decaying time as a function of pulse width
Supplementary Fig. 15. Additional XPS depth-profiling results of the Pt/WO_x/W memristors
Supplementary Fig. 16. XPS depth-profiling results for different sputtering condition.
Supplementary Fig. 17. Effects of the WO_x deposition condition on switching characteristics
Supplementary Fig. 18. Effects of different contacts and junctions on switching characteristics
Supplementary Fig. 19. Effects of different electrode combinations on switching characteristics
Supplementary Fig. 20. Visual inspection of the WO_x layer with different thickness
Supplementary Fig. 21. A switching curve and corresponding energy band diagrams
Supplementary Fig. 22. Effects of junction sizes on switching characteristics under vacuum
Supplementary Fig. 23. A X-ray diffraction analysis of the WO_x layer
Supplementary Fig. 24. Plots of current behaviors over time according to temperature
Supplementary Fig. 25. Switching characteristics using c-AFM technique
Supplementary Fig. 26. Area dependency
Supplementary Fig. 27. Physical reservoir states during 10 cycles for different pulse sequences
Supplementary Fig. 28. Physical reservoir states of different memristors at each layer
Supplementary Fig. 29. Final physical reservoir states for four possible cell positions
Supplementary Fig. 30. Physical reservoir states for five cell positions in a single reservoir

Supplementary Fig. 31. Prediction of time-dependent Lorenz attractor with a single reservoir

Supplementary Fig. 32. Prediction of time-dependent Lorenz attractor with multiple reservoir

Supplementary Fig. 33. Prediction of time-dependent Lorenz attractor with three multilayered perceptrons

Supplementary Fig. 34. Schematic examples of the hardware implementation at the circuit level

Supplementary Fig. 35. The additional cell images during the prediction processes

Supplementary Fig. 36. Simulated physical reservoir states of memristor cell

Supplementary Table

Supplementary Table 1. A summary of types of 3D stacked memristive arrays

Supplementary Note

Supplementary Note 1. Recent efforts on multilayered physical reservoir and the merit of our approach

Supplementary Note 2. Single and wide reservoir computing with WO_x memristor array

General reservoir computing

Single reservoir computing based on the WO_x memristors

Wide reservoir computing based on the WO_x memristors

Supplementary Note 3. Benefits of our approach compared to prior applications

Supplementary Note 4. 3D-integrated architecture

Supplementary Note 5. Potential switching mechanism of WO_x memristor

Potential switching mechanism

Electron trapping and detrapping processes during the switching

Minimum currents at non-zero voltage

Asymmetric profile of oxygen vacancies

Supplementary Note 6. Analyses and relevant discussions of wide physical reservoir computing based on multiple 3D stacked WO_x physical reservoirs

Requirements of the memristor for wide physical reservoir computing

Convergence speed for wide physical reservoir computing

Efficiency of wide physical reservoir computing compared to conventional approaches

Energy consumption for wide physical reservoir computing and conventional computing

Suggestion of circuit-level implementation of the physical wide reservoir computing

Supplementary references

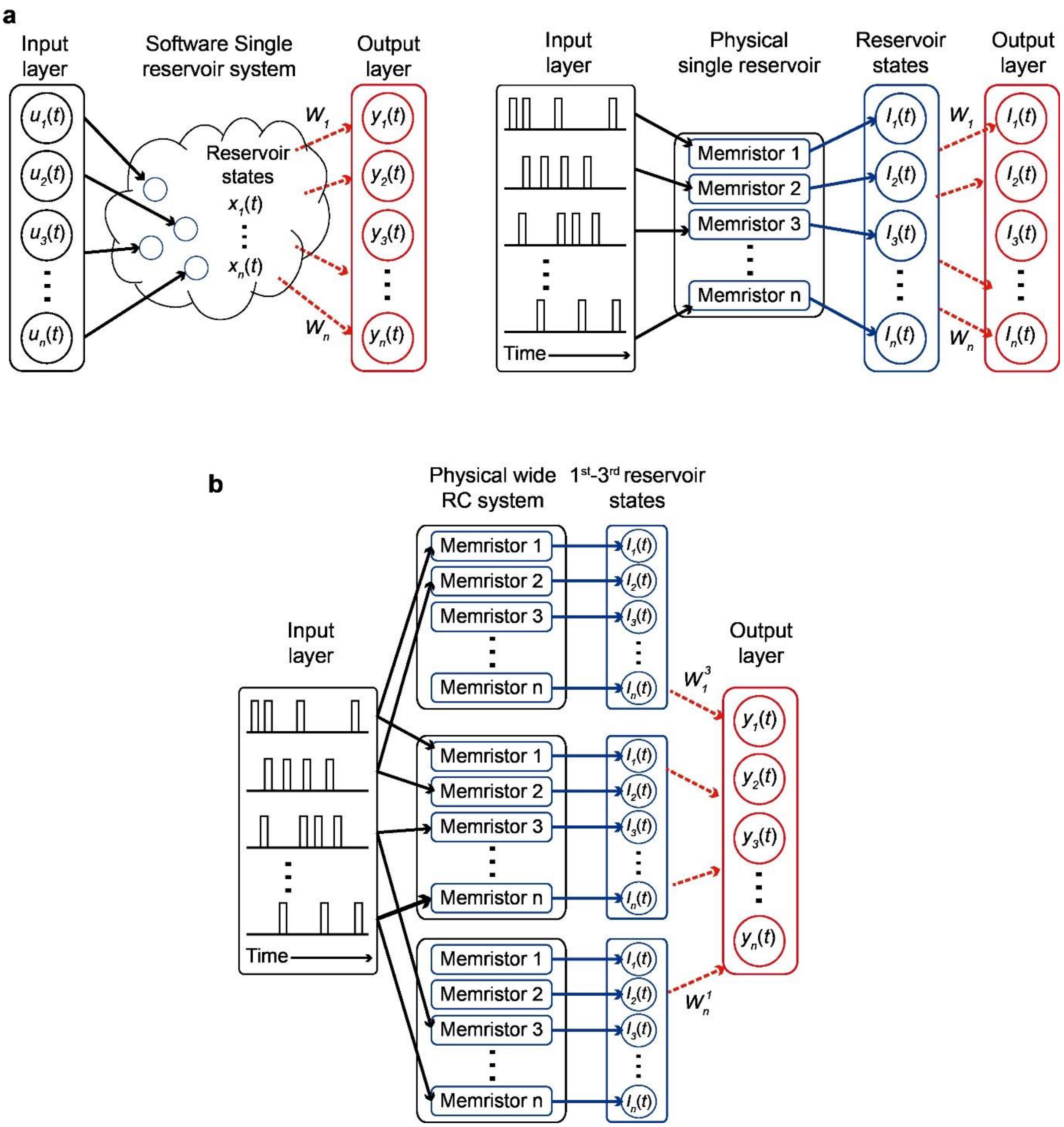

Supplementary Fig. 1. Conceptual schematics of the single and wide reservoir computing systems a, Software-based (left) and physical memristor-based single RC system (right). **b,** A physical memristor-based wide RC system. The detailed explanations are provided in Supplementary Note 2.

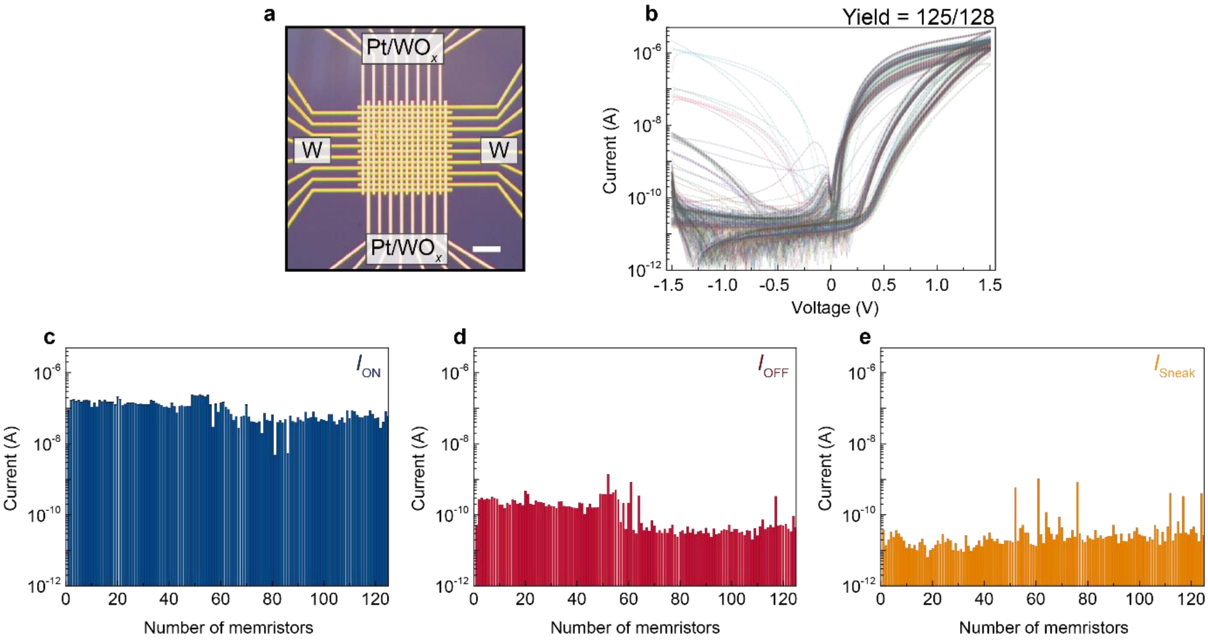

Supplementary Fig. 2. A 16 × 16 WO$_x$ memristive crossbar array with a line width of 20 μm a, A top-view optical microscopy (OM) image of the fabricated 16 × 16 crossbar array consisting of Pt/WO$_x$/W memristors. The scale bar is 200 μm. **b,** Overlapped self-rectifying *I*–*V* switching curves of the memristor cells (128 cells) in the array. The device yield was estimated to be 97.7 % (125/128), comparable to that of the 3D stacked array. **c-e,** Statistical histograms of I_{ON}, I_{OFF}, and I_{Sneak} with respect to the 125 memristors, confirming the uniform switching parameters.

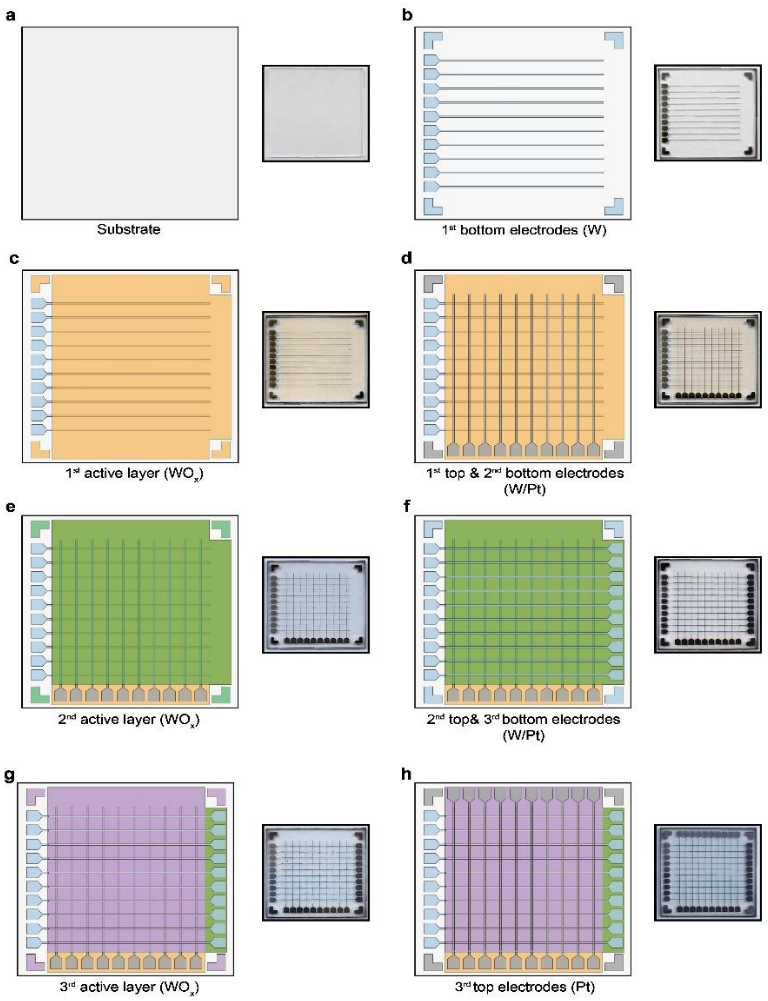

Supplementary Fig. 3. Schematic images for the fabrication procedure of the 3D stacked crossbar array and their corresponding OM images a, 1.5 cm × 1.5 cm glass substrate after the standard cleaning process. **b,** Formation of the 1st bottom W electrode lines with a 1.5 cm × 1.5 cm shadow mask. The 1st bottom W electrodes pads were positioned at the left side of the substrate. **c,** Deposition the 1st WO_x layer (orange) except for the left side of W electrode pads by using a shadow mask and RF sputter. The patterning align was carefully performed with the aid of OM. **d,** Formation of the 1st top Pt & 2nd bottom W electrode lines on the 1st WO_x layer with a shadow mask, perpendicular to the 1st bottom W electrode lines. The 2nd bottom W electrode lines were directly formed on the 1st top Pt electrode lines. The 1st top Pt & 2nd bottom W electrode pads were positioned at the lower part of the substrate. **e,** Deposition the 2nd WO_x layer (green) on the except for all the electrode pads by using a shadow mask and RF sputter. **f,** Formation of the 2nd top Pt & 3rd bottom W electrode lines on the 2nd WO_x layer with a shadow mask, perpendicular to the 1st top Pt & 2nd bottom W electrode lines. The 3rd bottom W electrode pads were positioned at the right side of the substrate. **g,** Deposition the 3rd WO_x layer (purple) on the except for all the electrode pads by using a shadow mask and RF

sputter. **h,** Formation of the 3rd top Pt electrode lines on the 3rd WO_x layer with a shadow mask, perpendicular to the 2nd top Pt & 3rd bottom W electrode lines. The 3rd top Pt electrode pads were positioned at the upper side of the substrate.

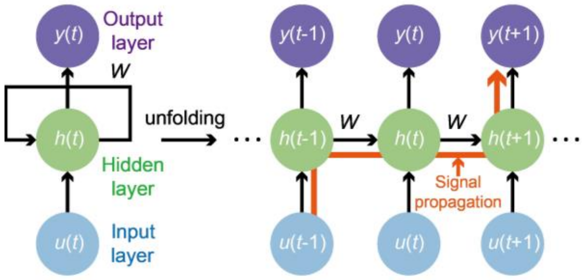

Supplementary Fig. 4. A Schematic of recurrent neural networks Certain $u(t+1)$ is fed to the network and its corresponding signal propagates to output layer (y) (black arrows). This process is influenced by previous states ($h(t\text{-}1)$ and $h(t)$) that remember the previous inputs to the network (orange arrow). Although this type of network is capable of producing outputs by utilizing sequential information, it requires intensive computations across timesteps and is vulnerable to gradient vanishing or exploding problems (i.e., $W \times W \times W\ldots$).

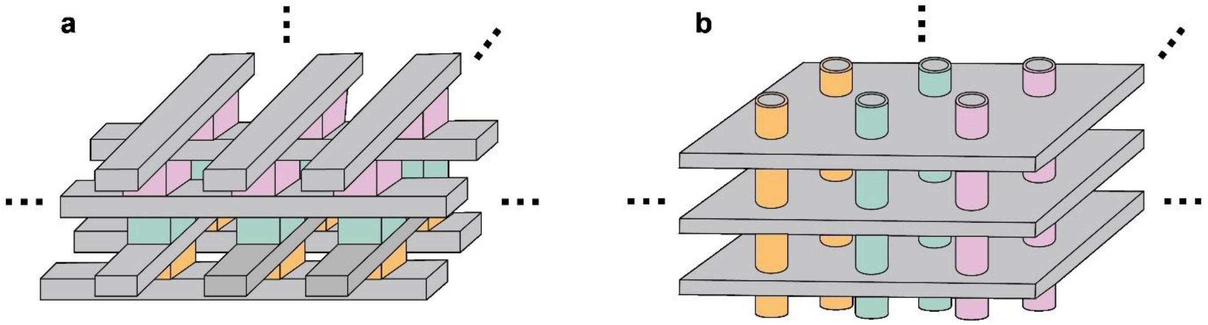

Supplementary Fig. 5. Schematics of 3D stacked memristive arrays a, b, horizontally (**a**) and vertically (**b**) stacked 3D architecture**.** Note that the illustrations represent standard architectures for each type.

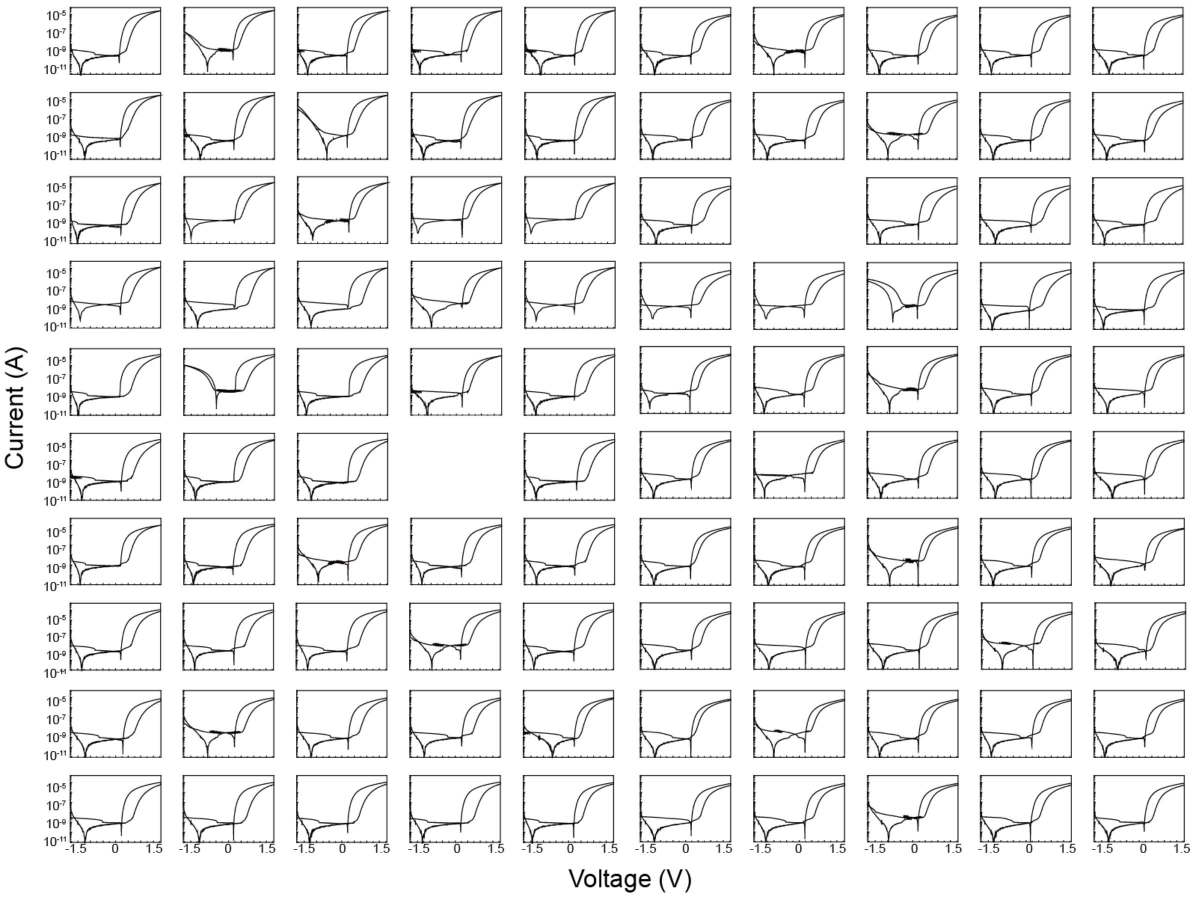

Supplementary Fig. 6. All self-rectifying switching curves for the 100 memristors at the first layer in the 3D stacked WO$_x$ PR arrays The switching failures with electrical short-circuit current behavior are represented as the blank (i.e., yield = 98 % (98/100)).

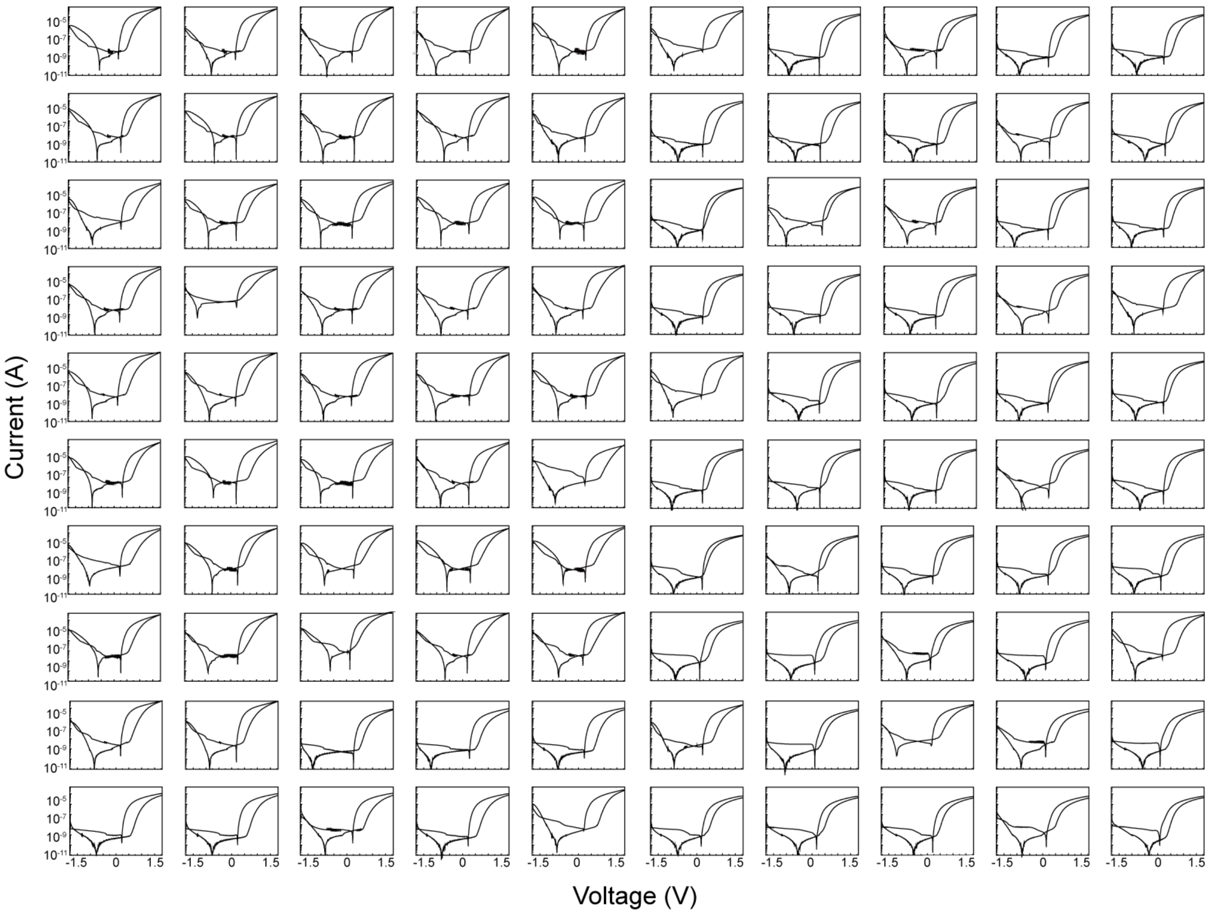

Supplementary Fig. 7. All self-rectifying switching curves for the 100 memristors at the second layer in the 3D stacked WO_x PR arrays The switching failures with electrical short-circuit current behavior are represented as the blank (i.e., yield = 100 % (100/100)).

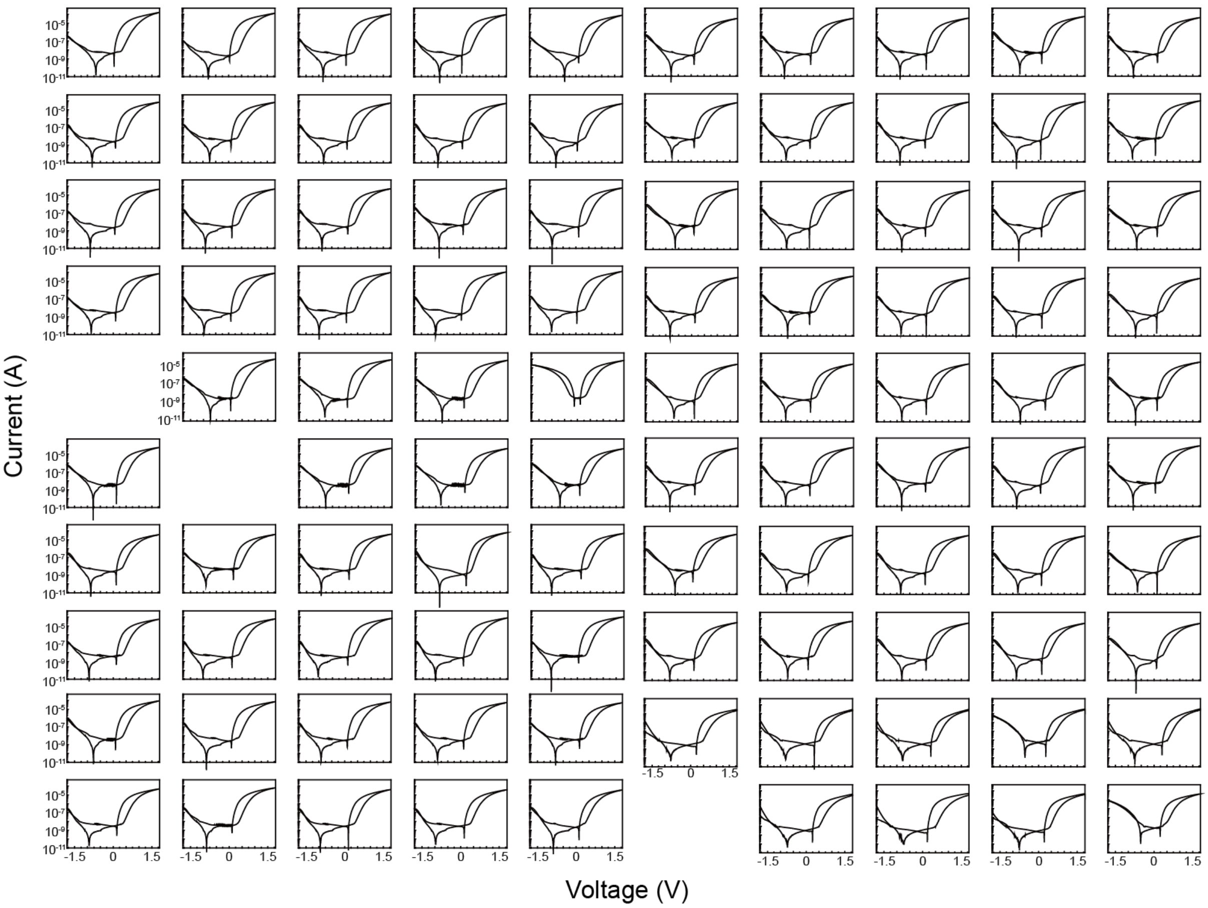

Supplementary Fig. 8. All self-rectifying switching curves for the 100 memristors at the third layer in the 3D stacked WO$_x$ PR arrays The switching failures with electrical short-circuit current behavior are represented as the blank (i.e., yield = 97 % (97/100)).

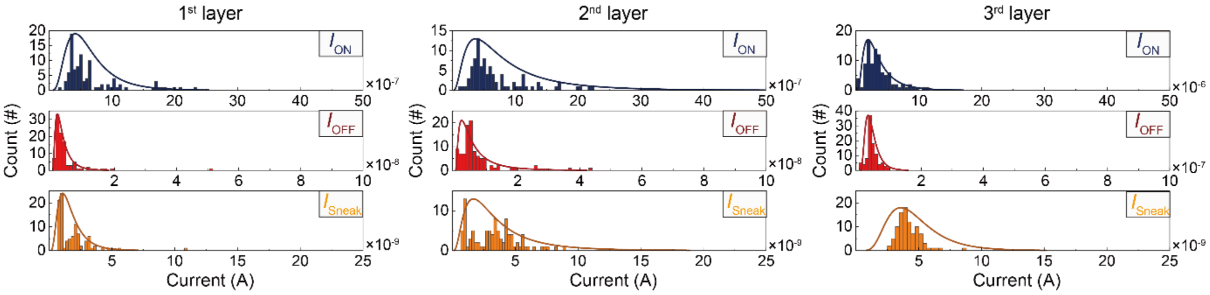

Supplementary Fig. 9. Statistical histograms of I_{ON}, I_{OFF}, and I_{Sneak} at each layer The switching parameters between memristors are well-fitted by the lognormal distribution curve. For the first layer, the mean (μ) and standard deviation (σ) of I_{ON}, I_{OFF}, and I_{Sneak} in natural logarithm scale (*ln*) are estimated to be -14.38 and 0.56, -19.68 and 0.73, and -20.32 and 0.58; for the second layer, -14.17 and 0.80, -19.15 and 0.94, and -19.65 and 0.75; for the third layer, -12.74 and 0.68, -17.08 and 0.52, and -19.25 and 0.44.

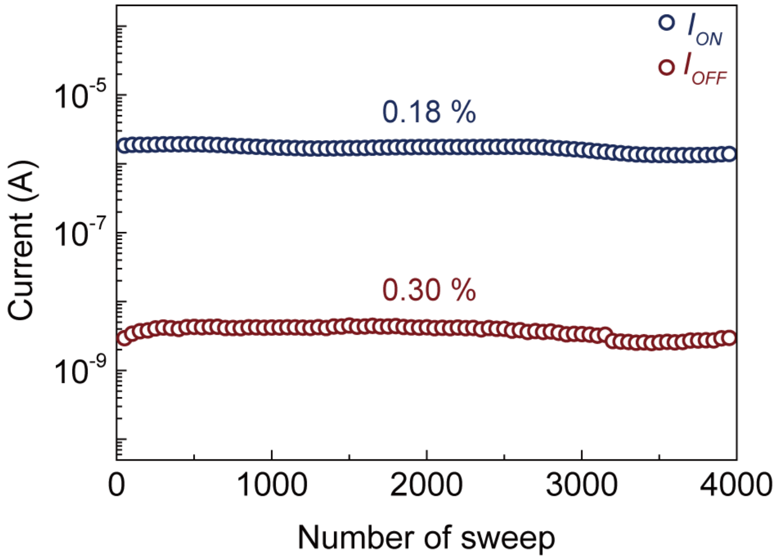

Supplementary Fig. 10. Cycle-to-cycle variation during the consecutive 4,000 sweeps The cycle variation for I_{ON} and I_{OFF} at the V_{READ} = 0.5 V was estimated by using coefficient of variation ($\sigma/\mu \times 100$ %). Note that μ is the average value and σ is the deviation. The μ and σ for I_{ON} were found to be 1.65×10^{-6} A and 3.63×10^{-9} A, whereas the μ and σ for I_{OFF} were found to be 2.93×10^{-9} A and 1.09×10^{-11} A. The average cycle-to-cycle variation was found to be ~0.24%.

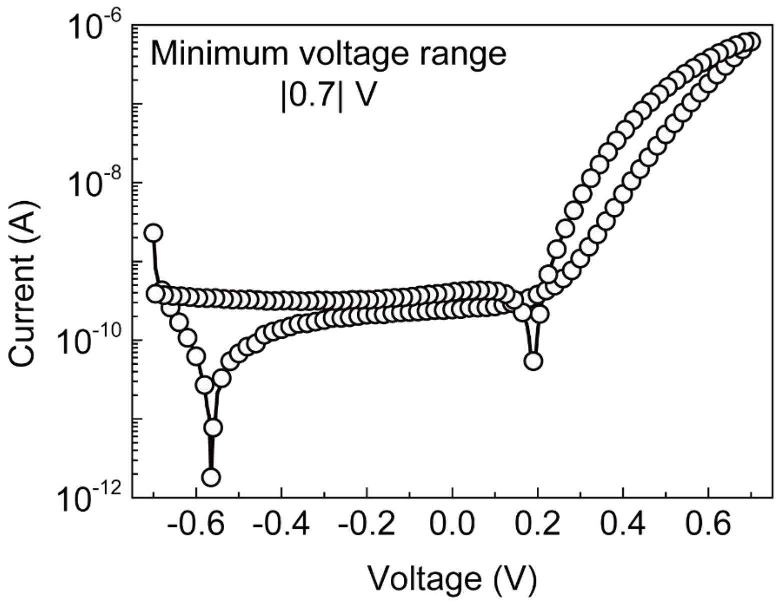

Supplementary Fig. 11. The lowest operating voltage range A selected self-rectifying *I*–*V* switching curve, exhibiting the lowest operating voltage range of ~0.7 V.

.

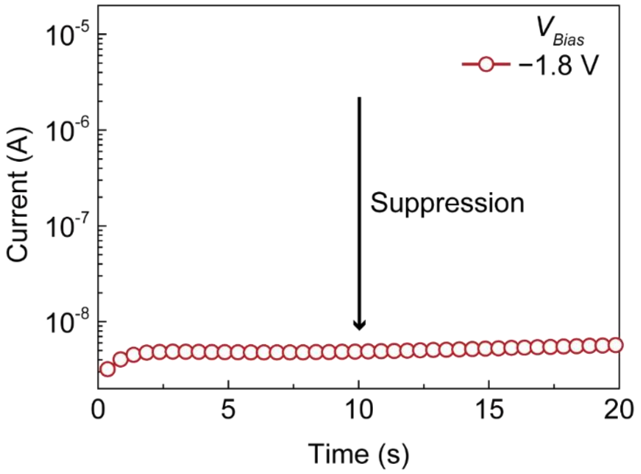

Supplementary Fig. 12. A plot of current-time for the self-rectifying property A current (*I*)–*time* plot at V_{Bias} = −1.8 V, showing the robust self-rectification.

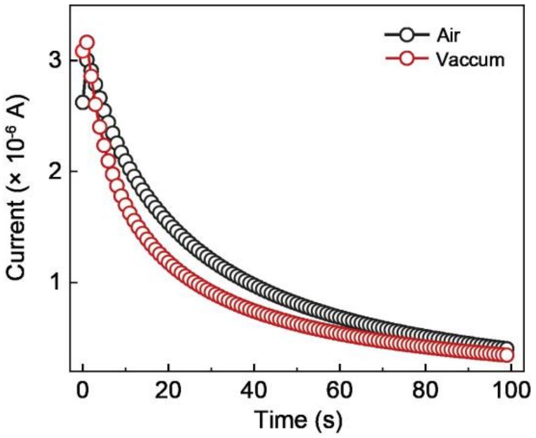

Supplementary Fig. 13. Plots of current behaviors over time under air and vacuum condition The current responses were read at V_{READ} = 0.5 V with time interval of 1 s after applying voltage sweep from 0 to 1.8 V. They all exhibit self-decaying characteristics despite the application of strong programming voltages, and there is almost no noticeable difference between the two conditions. Note that the vacuum is < 6 × 10^{-5} torr.

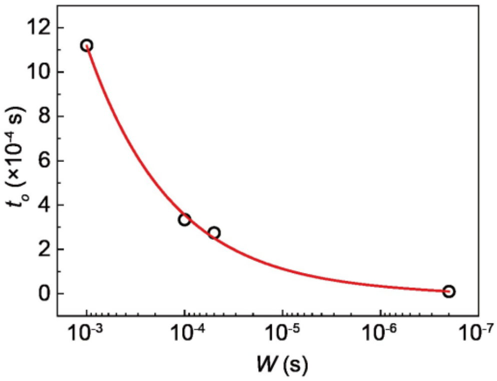

Supplementary Fig. 14. A plot of characteristics decaying time as a function of pulse width with the fitting curve of power function The analytic relation between characteristic decaying time (t_o) and pulse width (W) follows a power function (red line), expressed as:

$$t_o = A|W - x_c|^p \quad (1)$$

Note that the fitting parameters A, x_c, and p were found to be 3.49 × 10^{-2}, 1.21 × 10^{-7}, and 4.98 × 10^{-1}, respectively. Hence, we can conclude that the t_o can be tailored via the W according to the power function.

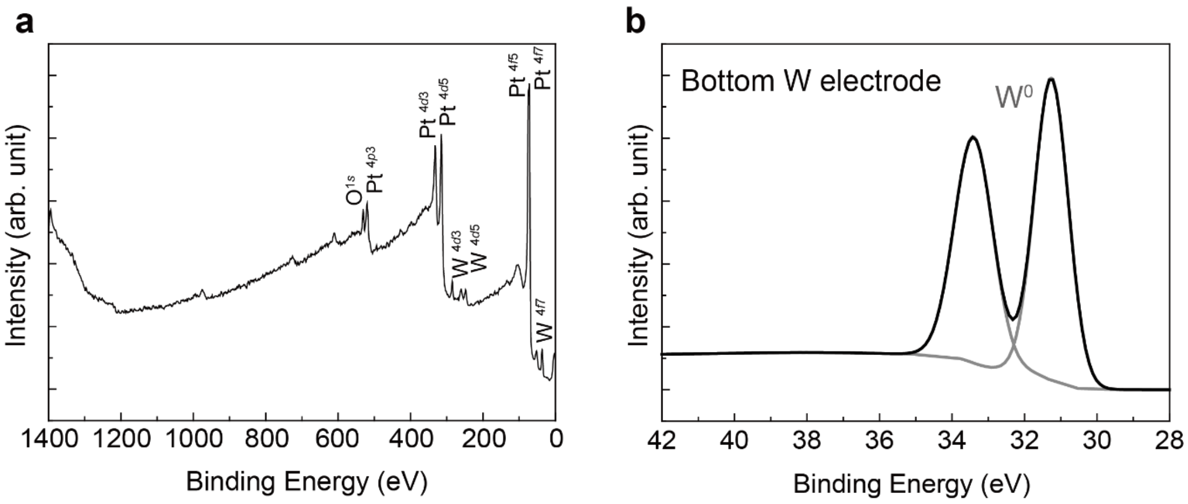

Supplementary Fig. 15. Additional XPS depth-profiling results to support the chemical composition and bonding states of the Pt/WO$_x$/W memristors a, A full-scan XPS spectra result of the fabricated Pt/WO$_x$/W memristor. Major peaks are observed to coincide with the binding energies of Pt, W, and O elements, confirming atomic elements in the junction structure. **b,** W 4*f* XPS results at the bottom W electrode. The two peaks coincided with the binding energy of the metallic W^0.

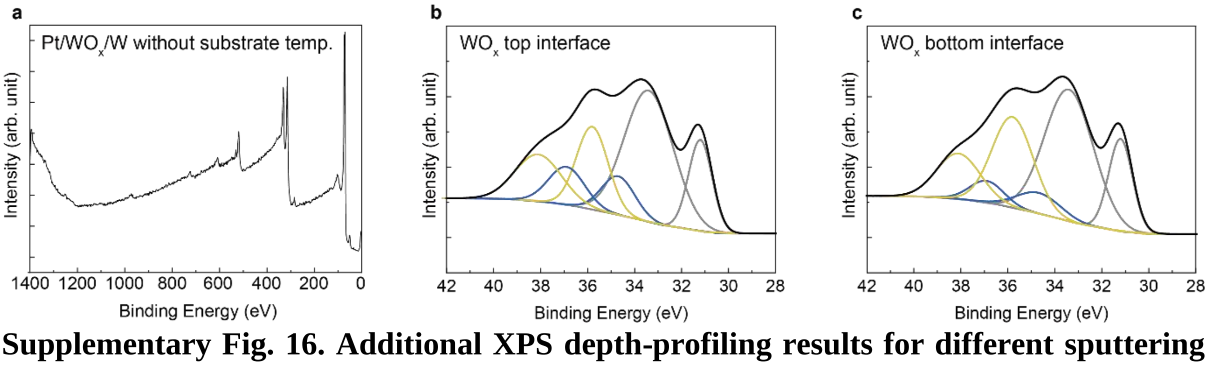

Supplementary Fig. 16. Additional XPS depth-profiling results for different sputtering condition a, Full-scan XPS spectra results. **b, c,** W 4*f* XPS results at the top (**b**) and bottom interface (**c**) for Pt/WO$_x$/W memristor without the substrate temperature.

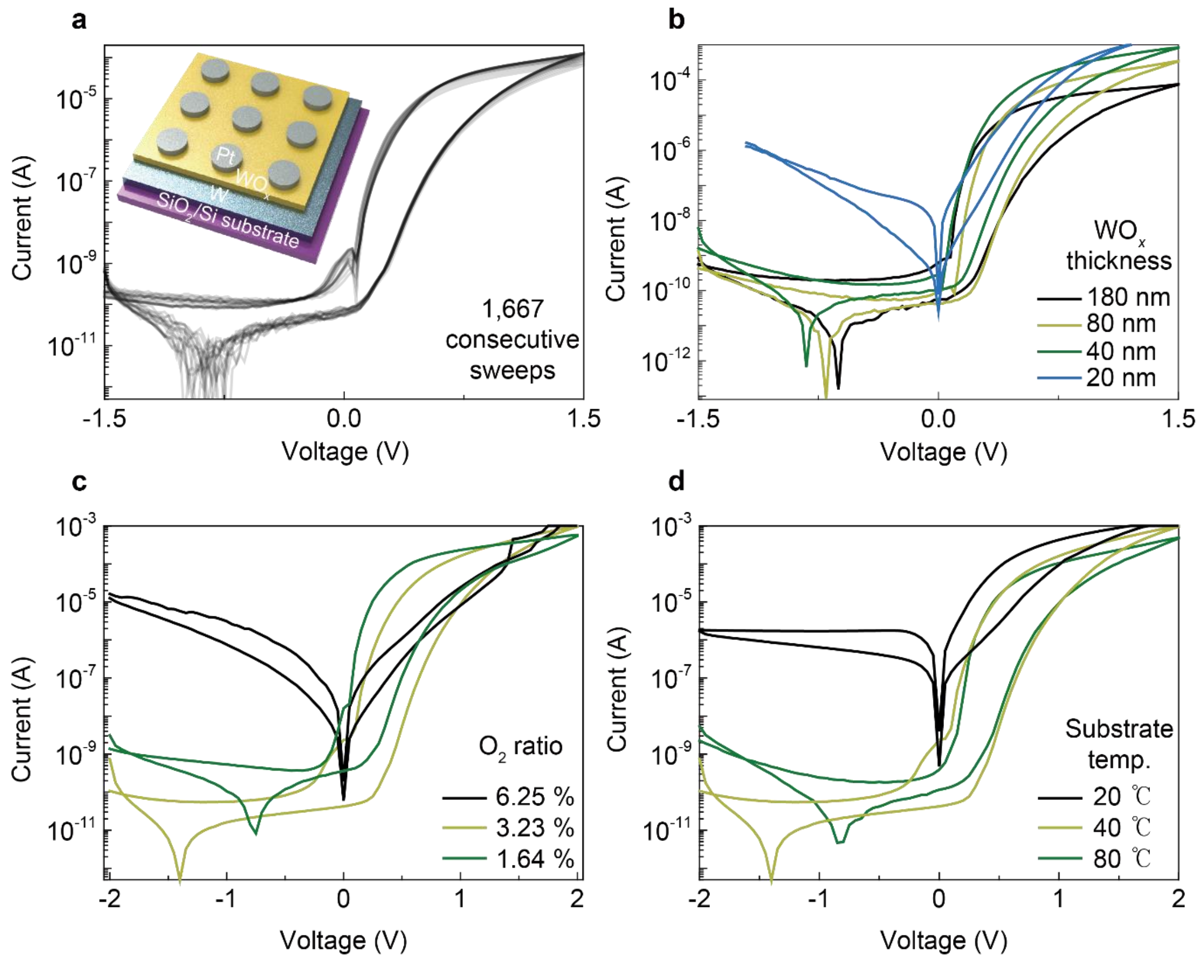

Supplementary Fig. 17. Change of switching characteristics of the Pt/WO$_x$/W memristors fabricated with different conditions of the WO$_x$ deposition a, Repeated *I*–*V* switching curves (1,667 times) of the Pt/WO$_x$/W memristor fabricated with identical experimental conditions used in this study (Fig. 2 and see Methods). The inset shows a schematic of the single WO$_x$ memristor cells. **b-d,** *I*–*V* switching curves of the WO$_x$ memristors as functions of the WO$_x$ thickness (from 20 to 180 nm) (**b**), O_2 ratio (from 1.64 to 6.25 %) (**c**), and the substrate temperature (from 20 to 80 °C) (**d**). These findings indicate that the sputtering parameters and substrate temperatures can determine the switching characteristics and can be used to design self-rectifying features.

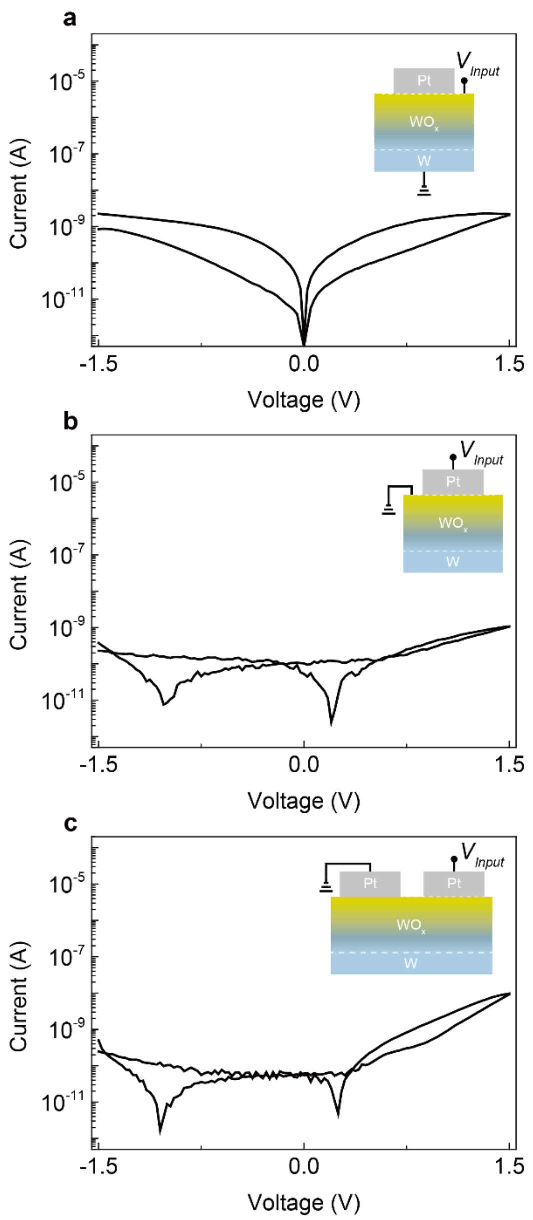

Supplementary Fig. 18. Electrical characteristics with different contacts and junction positions a-c, Representative *I*-*V* curves when contacting WO_x and W **(a)**, Pt and WO_x (**b**), and Pt and Pt contact (**c**) by the probing W tips. Observe that these results support two claims. The self-rectifying property was associated with the Pt/WO_x interfacial barrier, whereas the switching could be attributed to the WO_x/W barrier.

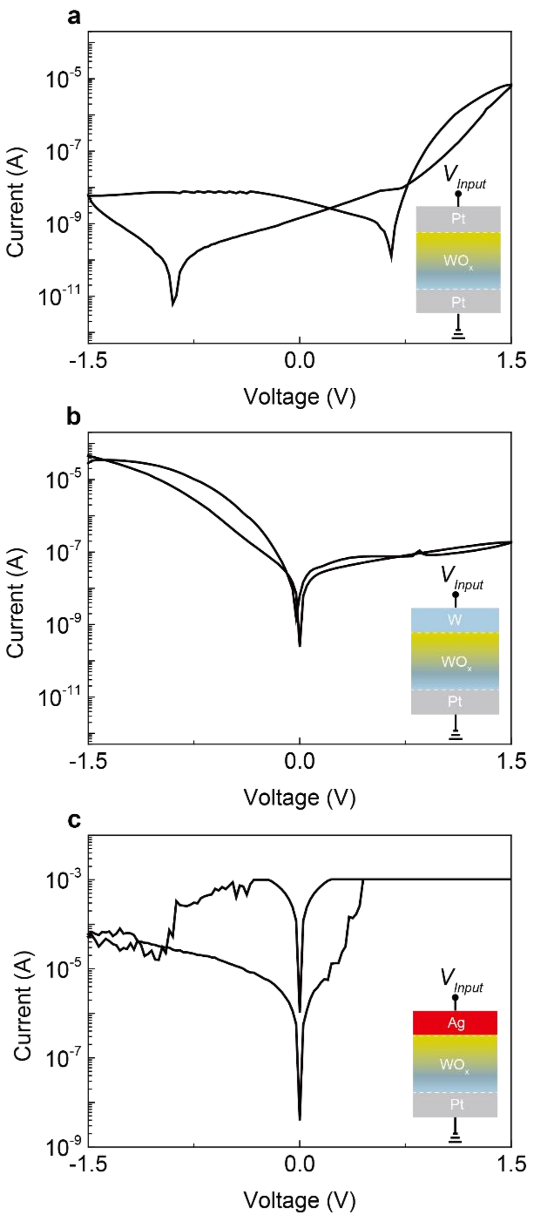

Supplementary Fig. 19. Electrical characteristics of WO_x junctions with different electrode combinations a-c, Representative *I*–*V* curves of Pt/WO_x/Pt (**a**), W/WO_x/Pt (**b**), and Ag/WO_x/Pt junction structures (**c**). These findings also support that self-rectifying switching feature is originated from both the Pt/WO_x and the WO_x/W interface barriers.

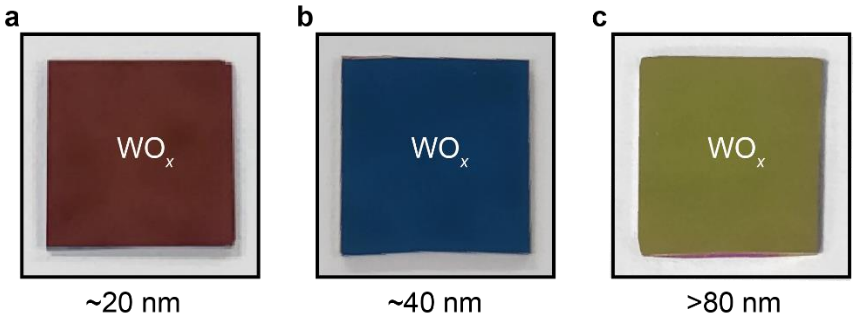

Supplementary Fig. 20. Visual inspection based on optical images of the top WO_x layer with different thickness a-c, Top-view optical images of the 20 nm (**a**), 40 nm (**b**), and >80 nm-thick WO_x layer (**c**) on the W/SiO_2/Si substrate. It is well-known that the yellow-, blue-, and brown-colored tungsten oxide indicate WO_3, WO_x ($2<x<3$), and WO_2, respectively.[1] Hence, these observations suggest and support the depth-dependent C_{V_O} of WO_x.

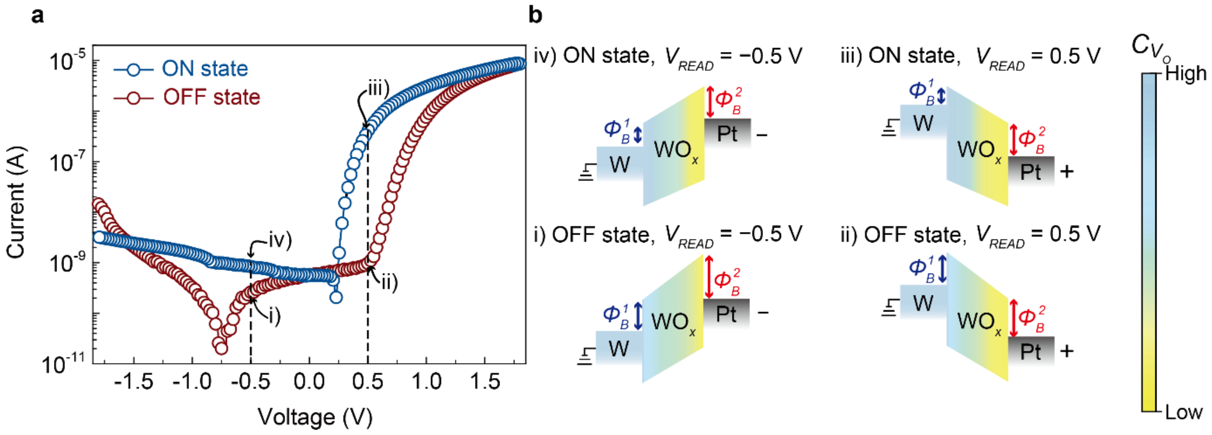

Supplementary Fig. 21. A self-rectifying *I*–*V* switching curve and corresponding energy band diagrams a, A *I*–*V* switching curve for the Pt/WO_x/W memristor with marked i)-iv) points where the *I*–*V* curves for ON and OFF state are represented as blue and red circles. **b,** The energy band diagrams for the i)-iv) points with the state-dependent C_{V_O} in the WO_x layer. Additional discussions are provided in Supplementary Note 5.

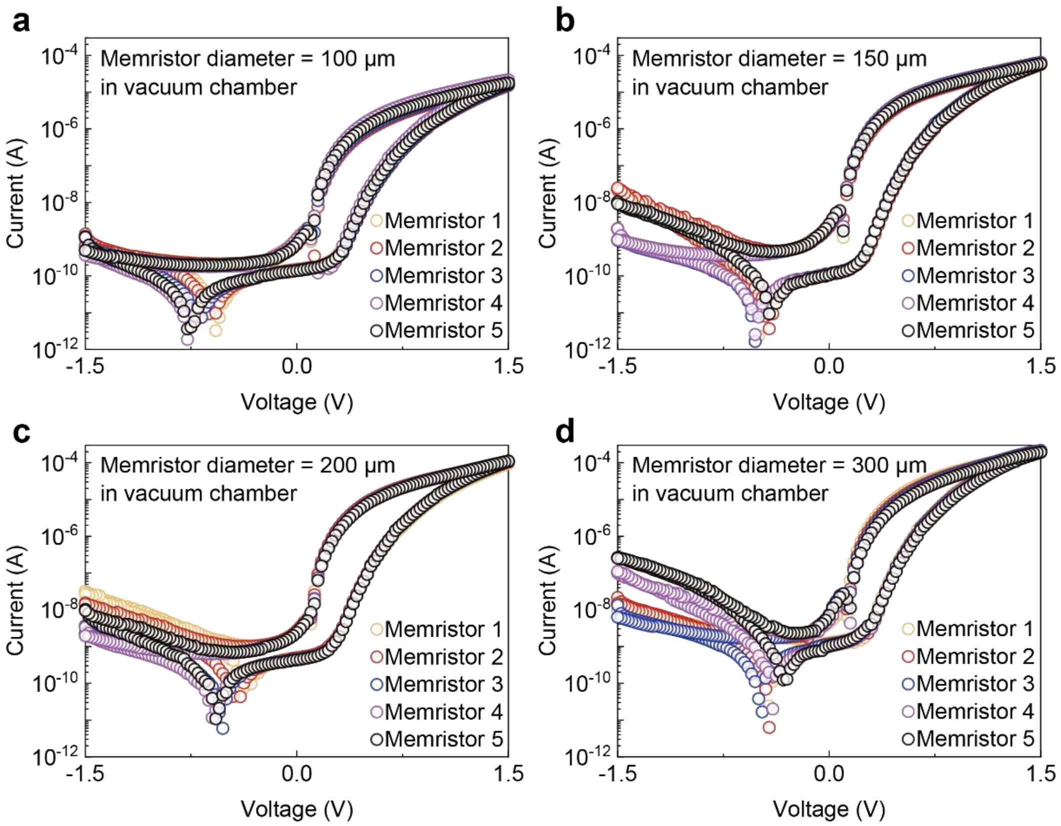

Supplementary Fig. 22. Electrical characteristics of the WO_x memristors as a function of junction diameter size under vacuum condition a-d, I–V switching curves of five Pt/WO_x/W memristors with 100 μm- (**a**), 150 μm- (**b**), 200 μm- (**c**), and 300 μm-diameter size (**d**) under vacuum condition (< 10^{-5} torr). They all exhibit robust switching behaviors regardless of the junction size. Note that there is no noticeable difference between air and vacuum environments. This result might undermine the possibility of an H-mediated switching mechanism originated from the air.[2]

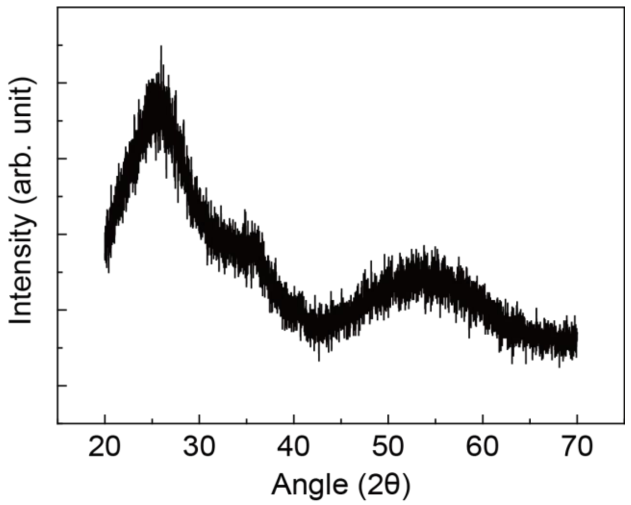

Supplementary Fig. 23. A X-ray diffraction analysis of the WO_x layer Broad diffraction peaks instead of sharp peaks are observed, indicating the amorphous phase with very small crystallites in the short-range order.[3]

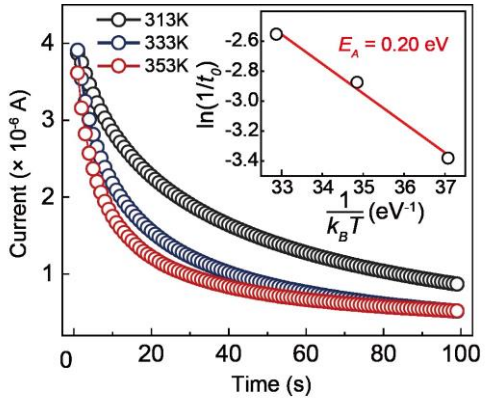

Supplementary Fig. 24. Plots of current behaviors over time as a function of temperature The current responses were read at V_{READ} = 0.5 V with time interval of 1 s after applying voltage sweep from 0 to 1.8 V. The inset shows the Arrhenius plot of ln(1/t_o) according to 1/k_BT. The E_a was estimated from the slopes of the straight fitting lines shown in the inset.

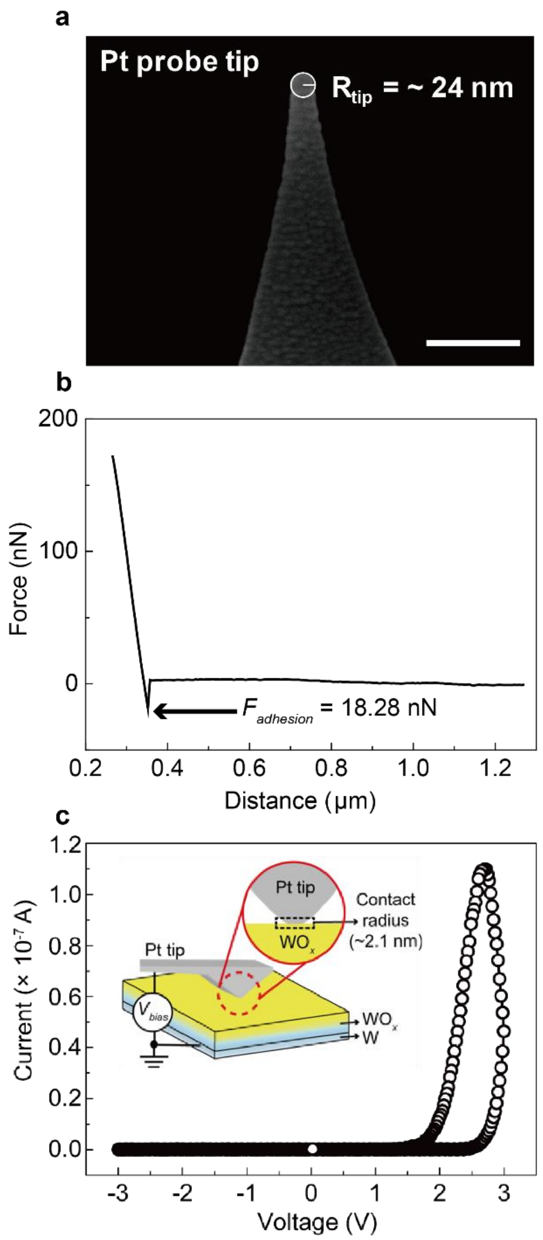

Supplementary Fig. 25. The *I*–*V* switching feature at the nanoscale using conductive atomic force microscopy technique a, A scanning electron microscopy (SEM) image of top-view for a nanoscale Pt tip. The radius of the Pt tip (R_{tip}) was found to be ~24 nm. The contact radius ($R_{contact}$) is estimated to ~2.1 nm. The scale bar is 200 nm. **b,** Investigation of the adhesion force ($F_{adhesion}$) during the contact. **c,** A *I*–*V* switching curve of the selected position on the WO_x/W sample with Pt tip. The inset shows a schematic illustration of the conductive atomic force microscopy (c-AFM) measurement, exhibiting the same junction structure as the Pt/WO_x/W memristor of the 3D stacked array.

To estimate the contact radius ($R_{contact}$) during the c-AFM measurement, Hertzian elastic contact model was utilized, expressed as[4]:

$$R_{contact} = (R_{tip} \cdot F_n / K)^{1/3} \qquad (2)$$

Note that F_n is net force determined by the sum of tip-loading force ($F_{loading}$ (10 nN)) and $F_{adhesion}$ (18.28 nN), and K is elastic modulus of WO_x (~70 GPa)[5]. Consequently, the estimated $R_{contact}$ was found to be ~2.1 nm. As shown in Supplementary Fig. 25c, a similar self-rectifying switching behavior was observed with the ~2 nm contact area, confirming the nanoscale switching.

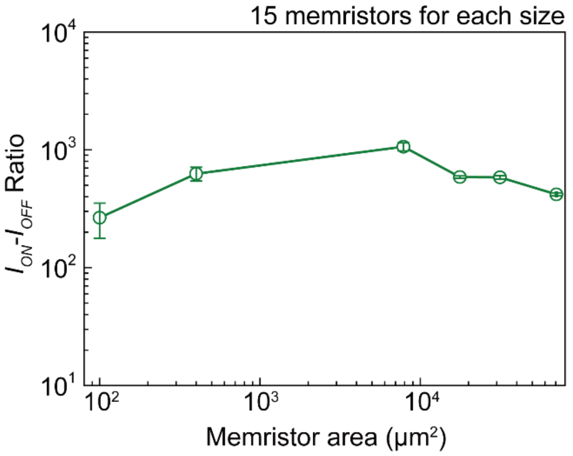

Supplementary Fig. 26. A plot of I_{ON}/I_{OFF} ratio of 15 memristors as a function of memristor area Despite the decrease in the size, I_{ON}/I_{OFF} was observed to be almost maintained, supporting the homogeneous interfacial switching.

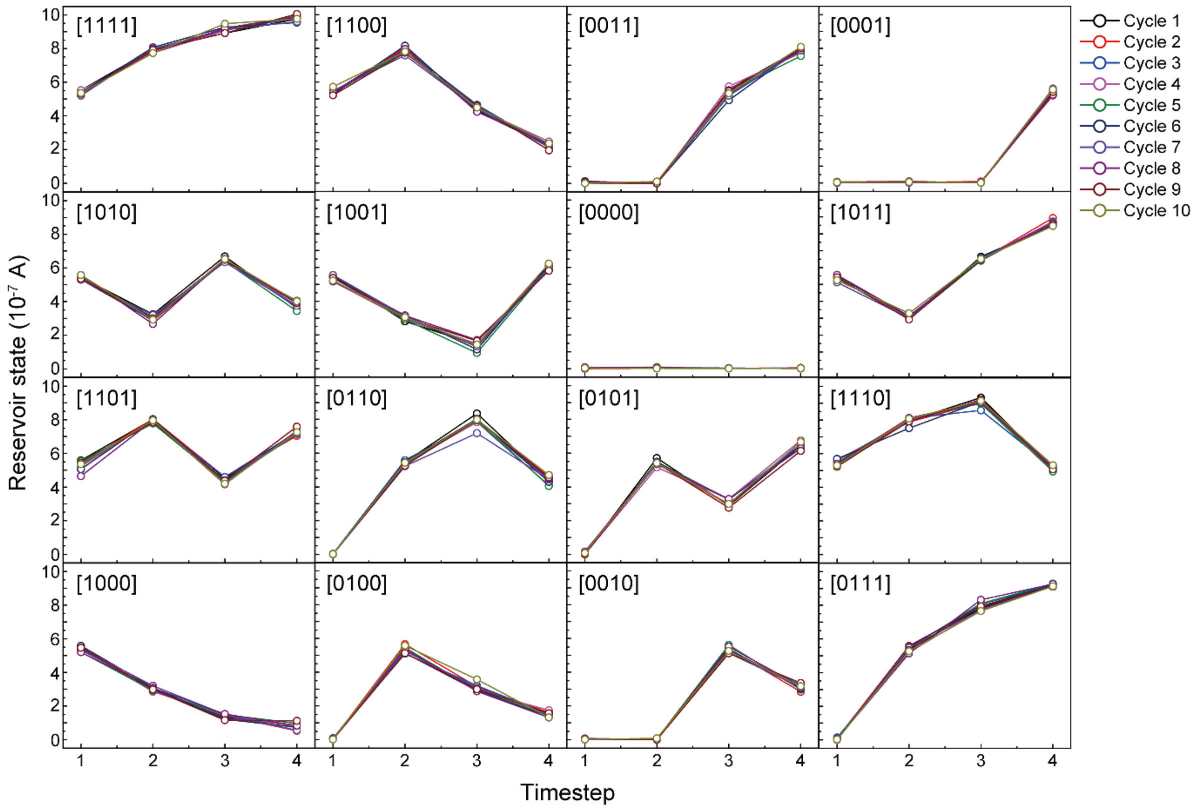

Supplementary Fig. 27. Change in physical reservoir states of the selected memristor Physical reservoir (PR) states were recorded at V_{READ} = 0.5 V per timestep (t_1-t_4) during 10 cycles with respect to 16 voltage pulse sequences**,** demonstrating the uniform and distinct PR states.

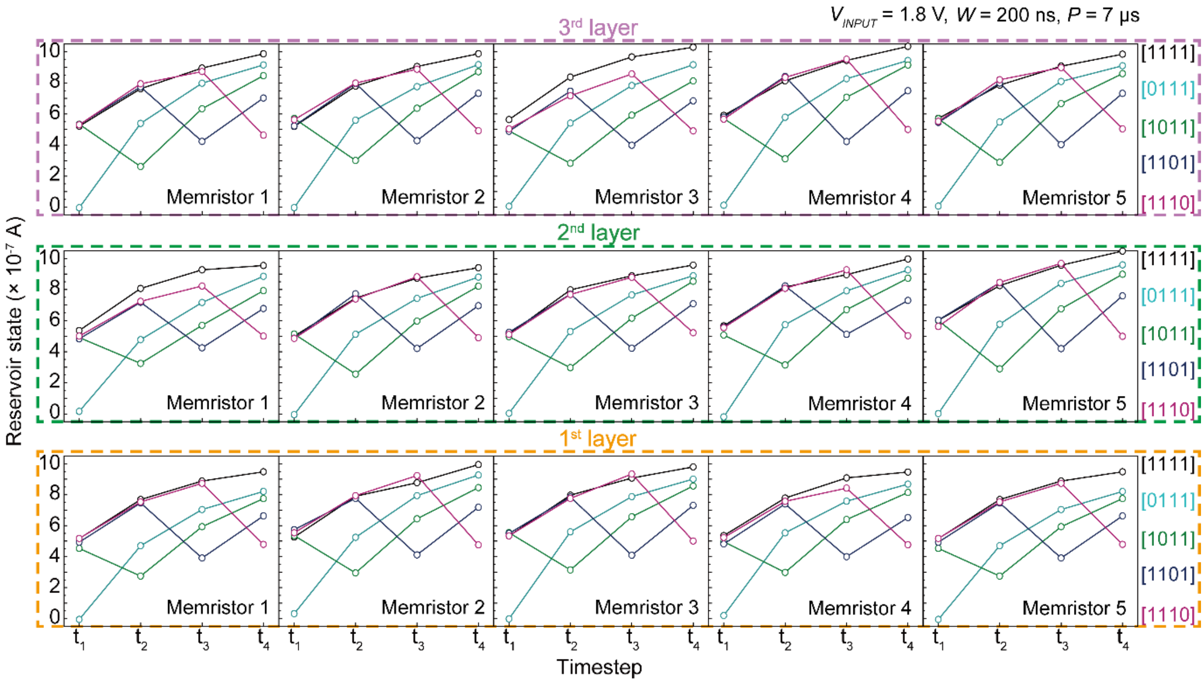

Supplementary Fig. 28. All the plots of physical reservoir states of different memristors at each layer in the 3D stacked WO_x PRs array. Change in PR states of the selected five memristor of first (lower, orange), second (middle, green), and third layer (upper, purple) at V_{READ} = 0.5 V per timestep (t_1-t_4) with respect to certain voltage pulse sequences. They all exhibit similar trajectories of change in the PR states of each layer over timestep and small device variation. Note that despite the same time-dependent inputs, quantitatively different PR states that share qualitative similarities could be obtained due to the small device variation, which could further enrich the reservoir states of the 3D stacked array.

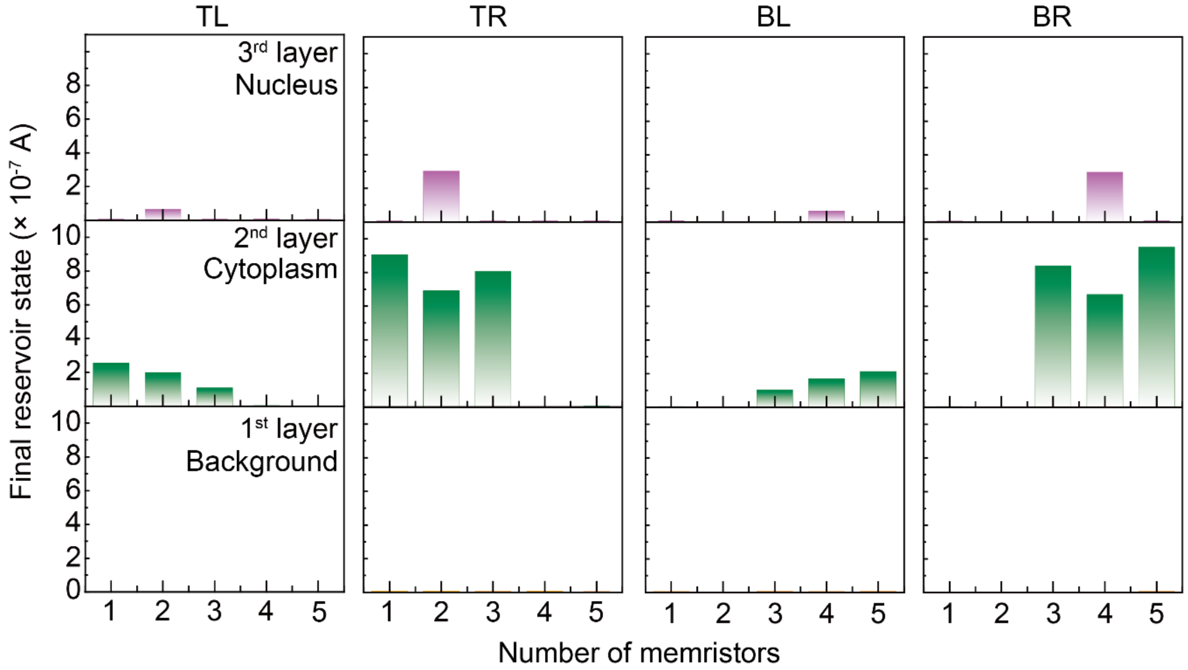

Supplementary Fig. 29. All the plots of final physical reservoir states of five memristors per layer for four possible cell positions For TL, TR, BL, and BR, different PR states were achieved at t_5. Note that C is shown in Fig. 4e. Each reservoir can effectively capture the spatiotemporal information from the local features, which could facilitate the learning process at the output layer.

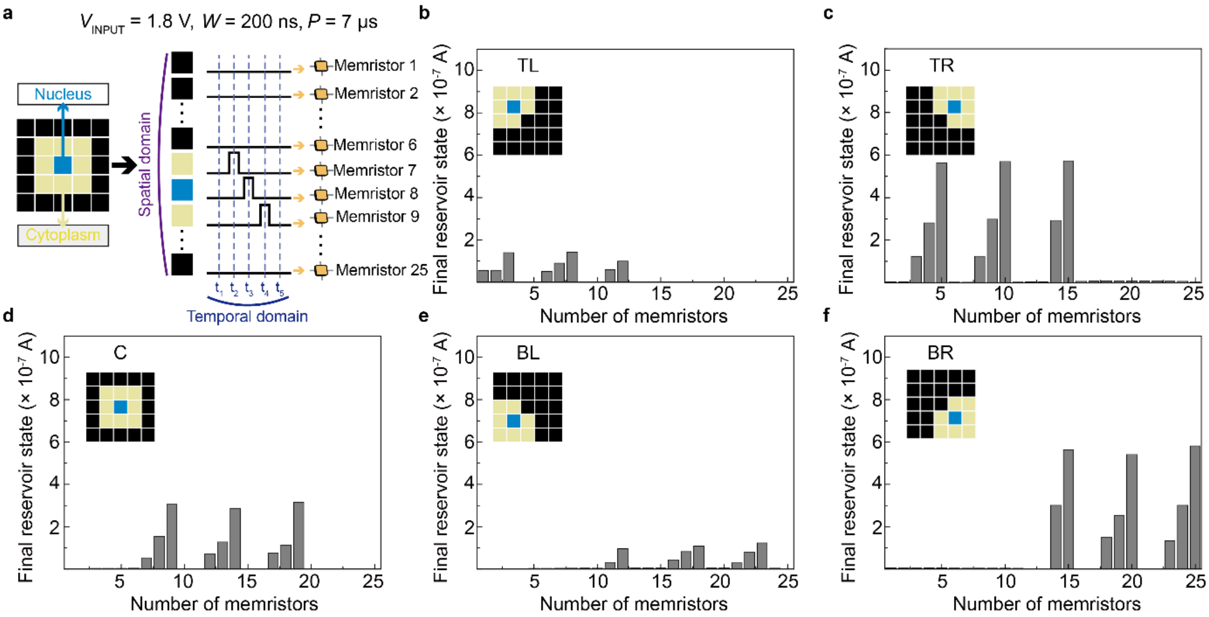

Supplementary Fig. 30. Physical reservoir states for five cell positions in a single reservoir

a, Schematics of a 5 × 5-pixel image for case C, and their corresponding time-series sequences applied to the single reservoir consisting of 25 memristors. **b-f,** Final reservoir states at t_5 obtained from the single reservoir (25 memristors) for TL (**b**), TR (**c**), C (**d**), BL (**e**), and BR (**f**).

For unbiased comparison, 25 memristors were used within the single reservoir to extract spatiotemporal information of the different cell images, considering that reservoir capacity can be also improved by increasing the number of memristors. As shown in Supplementary Fig. 30b-30f, nevertheless, multiple local features were hardly separated when using the single reservoir. For example, the single reservoir was not able to distinguish the nucleus and cytoplasm features due to the overlapping position, making it hard to classify different cell positions. This is mainly because one of the key parameters to detect the cell position is to identify the nucleus position. In contrast, multiple reservoirs could address the individual traits of the multiple time-series information, which could readily perform a detection task of the moving biological cells.

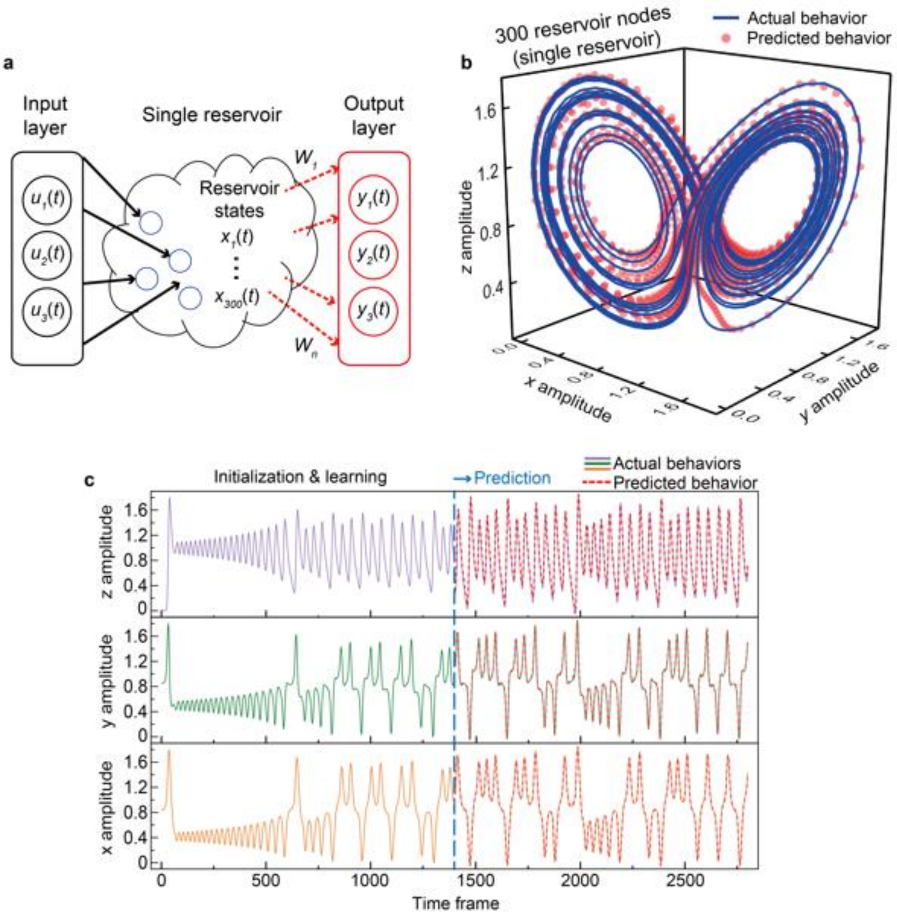

Supplementary Fig. 31. Prediction of time-dependent Lorenz attractor with a single reservoir a, Schematic of a single reservoir system with 300 memristors. **b,** Actual and predicted behaviors of 3D Lorenz attractor based on the single reservoir. **c,** Individual actual behaviors of *x*, *y*, and *z* components over time (colored solid lines), and their corresponding prediction results (red dotted lines) using the single WO_x PRs after 1,400 timesteps (blue dotted line). Average NMSE between actual and predicted behaviors at 2,788 timestep was estimated to be 1.35×10^{-3}, which was ten-fold worse than the use of multiple reservoirs consisting of the same number of memristors (See, Supplementary Note 2 and 6).

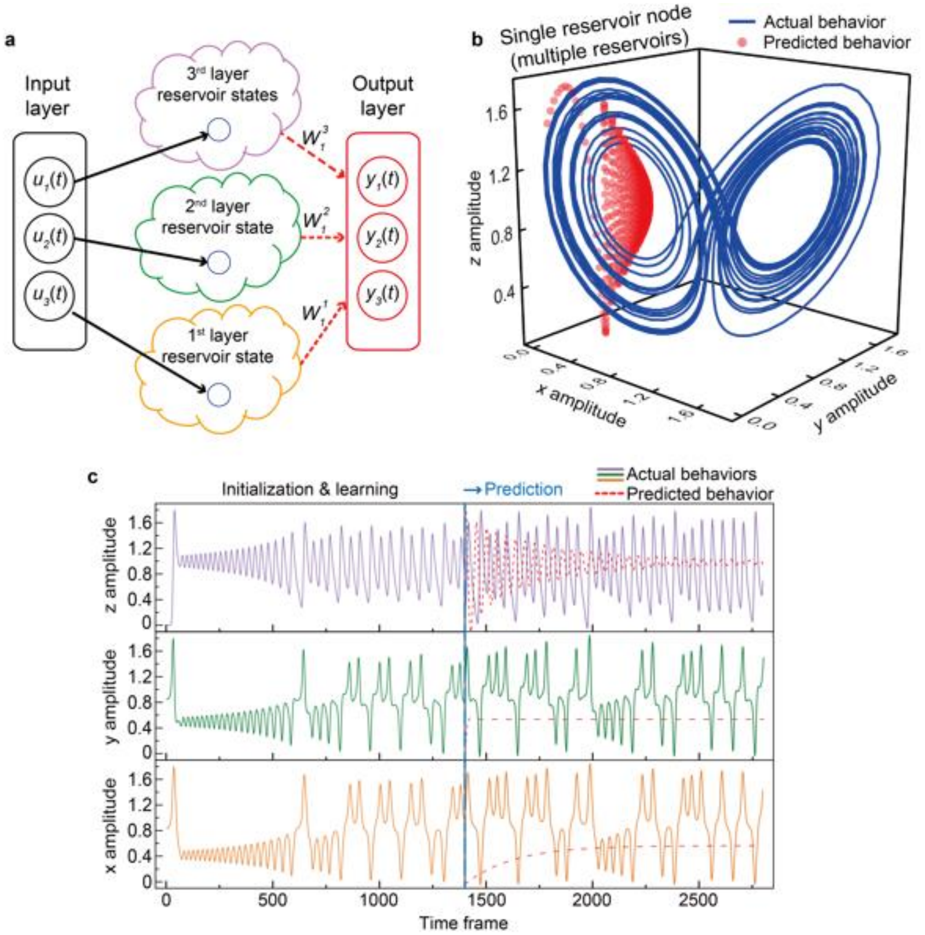

Supplementary Fig. 32. Prediction of time-dependent Lorenz attractor with multiple reservoirs a, Schematic of a multiple reservoir system with 1 memristor per layer. **b,** Actual and predicted behaviors of 3D Lorenz attractor. **c,** Individual actual behaviors of *x*, *y*, and *z* components over time (colored solid lines), and their corresponding prediction results (red dotted lines) using the single WO_x PRs after 1,400 timesteps (blue dotted line). Due to the insufficient reservoir capacity, the prediction of the Lorenz attractor was failure. Further discussions are provided in Supplementary Note 2 and 6.

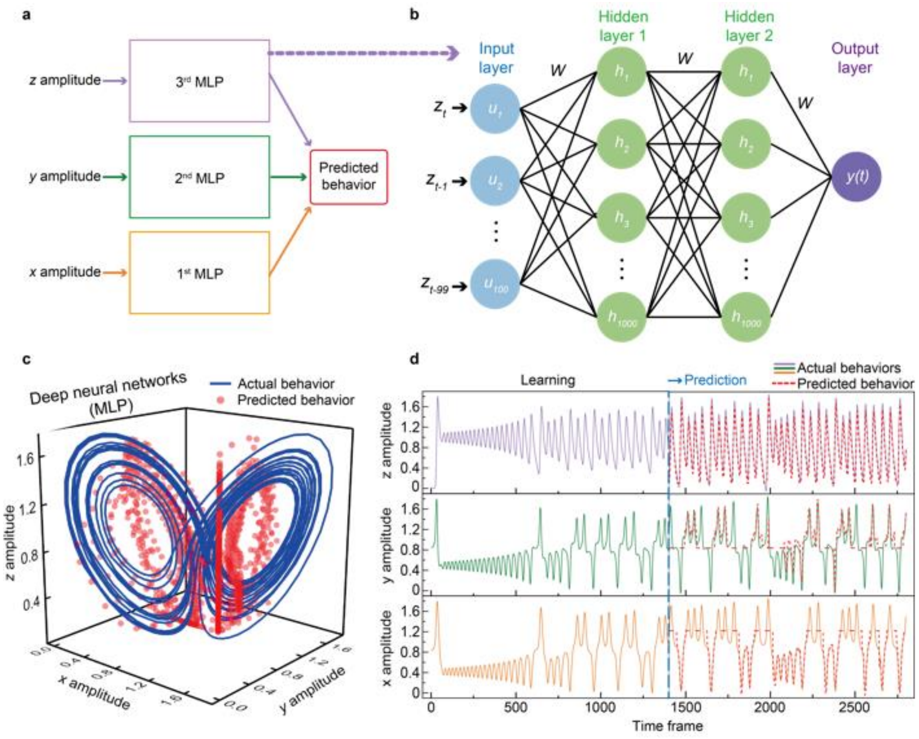

Supplementary Fig. 33. Prediction of time-dependent Lorenz attractor with multilayered perceptrons a, A schematic of three multilayered perceptrons (MLPs) for prediction of the 3D Lorenz attractor. **b,** An example of 100-1000-1000-1 network configuration for the 3rd MLP. Note that bias is omitted for simple illustration. **c,** Actual and predicted behaviors of 3D Lorenz attractor. **d,** Individual actual behaviors of *x*, *y*, and *z* components over time (colored solid lines), and their corresponding prediction results (red dotted lines) using the three MLPs after 1,400 timesteps (blue dotted line). The prediction of the Lorenz attractor was unsuccessful, which might be related to the insufficient capability of capturing temporal correlation between sequential inputs (Supplementary Note 6).

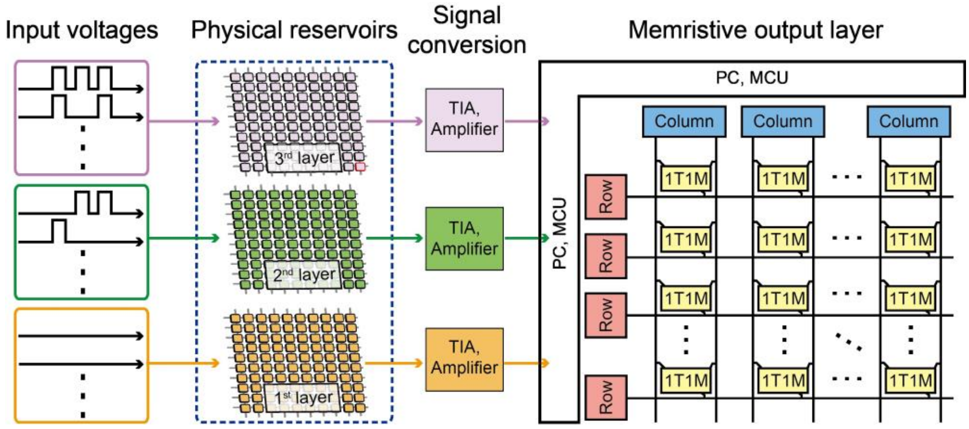

Supplementary Fig. 34. A **Suggestion of circuit-level implementation** Schematic examples of the hardware implementation of the physical wide RC at the circuit level.

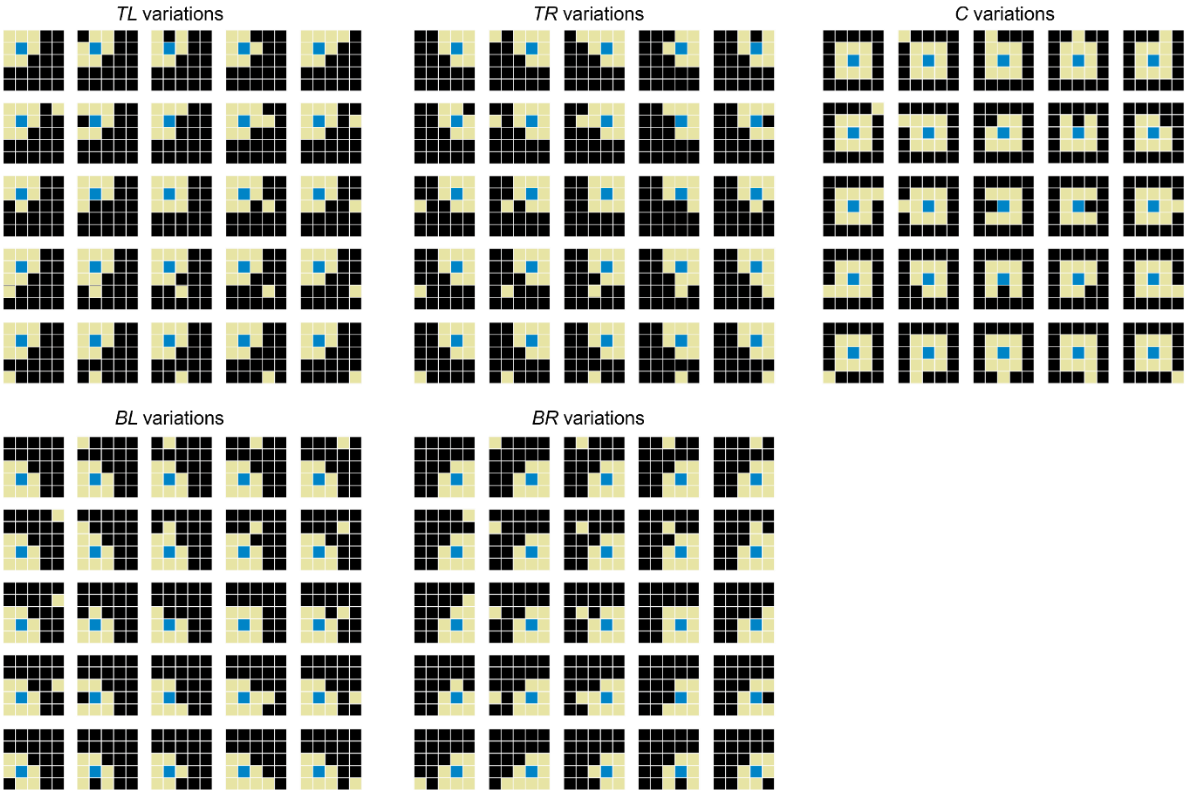

Supplementary Fig. 35. The additional cell images during the prediction processes The learning images per cell position (Fig. 4d), generated by using one-pixel flip method, were also included during the prediction.

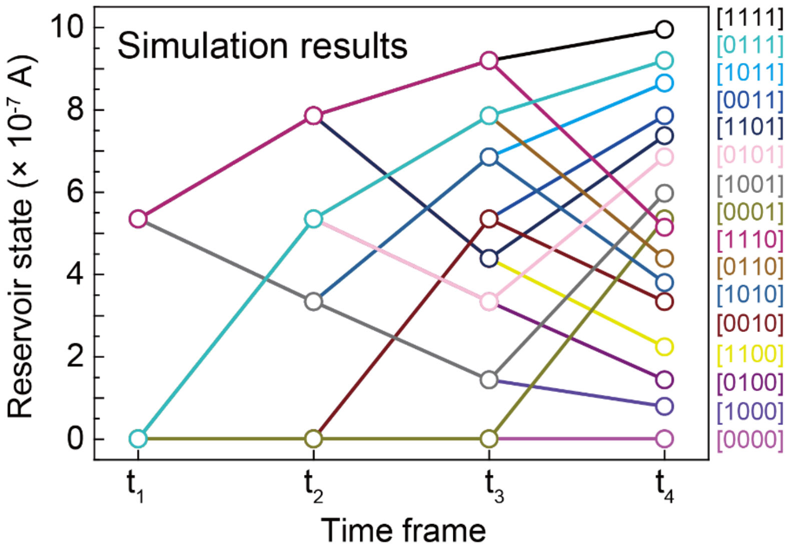

Supplementary Fig. 36. Simulated physical reservoir states of memristor cell Physical **r**eservoir states are simulated and plotted based on nonlinear behaviors of memristor per timestep and 16 voltage pulse sequences.

Because current behaviors were observed to nonlinearly change according to input voltages, the reservoir states ($r(t)$) could be fitted by using exponential equations:

$$r(t) = r(t\text{-}1) + \Delta r(t\text{-}1) \tag{3}$$

where $\Delta r(t\text{-}1) = a + be^{-c\frac{r(t-1)-I_{OFF}}{I_{ON}-I_{OFF}}}$ and $d + fe^{-g\frac{I_{ON}-r(t-1)}{I_{ON}-I_{OFF}}}$ for presence and absence of input voltage at a prior timestep (t-1), respectively. Note that a, b, c, d, f, and g are fitting parameters. Because $\Delta r(t\text{-}1)$ is determined by the $r(t\text{-}1)$, different $r(t)$ dependent on the temporal voltage input sequences could be generated and simulated as shown in Supplementary Fig. 36. Based on the fitting results, the prediction processes of the wide physical RC were performed.

Type / Feature	Horizontal type	Vertical type
Scalability	Scalable Laterally	Scalable Vertically
Individual integration	Feasible	Infeasible
Cost (for vertical direction)	Inefficient (Critical lithography $\propto$ the stacked layer)	Efficient (a smaller number of critical lithography)
Density	High	High
Fabrication complexity	Simple fabrication processes	Complex fabrication processes

Supplementary Table 1. A summary of horizontal and vertical types of 3D stacked memristive arrays with major features All parameters are represented the relative features to each other. For example, the fabrication cost of the horizontal type is relatively high than that of the vertical types.

SUPPLEMENTARY NOTES

Supplementary Note 1. Recent efforts on multilayered physical reservoir and the merit of our approach

There have been a few reports regarding the multilayered physical reservoir. First, Liu, K. et al demonstrated a multilayer RC based on three-terminal ferroelectric devices and 2D frameworks.[6] They laterally connected three-terminal ferroelectric devices to form a two-layer reservoir system and predicted electrical energy consumption in Brazil and triangle/square waveform classification tasks. Since temporal inputs unidirectionally propagate from the first reservoir device to second reservoir device, the first reservoir could process high-frequency inputs while the second reservoir could handle low-frequency inputs. Accordingly, this multilayer RC system is appropriate to capture multi-scale dynamics of time-series data. This work suggested another multilayered reservoir architecture that can expand reservoir capacity based on 2D device frameworks. In addition, Sun et al reported a 3D deep RC system based on the pillar-type vertical structure with planar electrodes,[7] where each memristor acts as a reservoir following the concept of virtual nodes, and each layer receives all the x, y, and z components of temporal inputs. Different features of gesture inputs could be extracted by using three input strategy designs, expanding the reservoir dimensionality for a higher RC performance. This work contributed to the development of multilayer reservoir computing based on the interplay between input masking methods and vertical pillar structure.

Nevertheless, in terms of 3D-integrated reservoir computing array architecture and its components, our device system can be largely differentiated. For example, we constructed a 3D physical wide reservoir architecture by vertically stacked memristive crossbar arrays utilizing selector-less WO_x memristors, not based on laterally connected three-terminal ferroelectric devices in 2D framework or vertical pillar structure.

Herein, each layer functions as a reservoir and all memristors at each layer can generate different reservoir states with respect to the individual components of the multi-variable temporal inputs. In contrast to prior efforts, our approach can facilitate time processing in parallel regardless of the number and location of the stacked layers and devices because it can significantly mitigate the signal distortion from the combination of the many shared planar electrodes/pillars and the leakage currents. This is beneficial to the generation of consistent PR states response to the identical temporal inputs, thus ensuring the separation property of the wide PR arrays. Moreover, the additional processing steps such as input mask design and

virtual nodes could be minimized, which makes peripheral circuits simpler and reduces their relevant time/energy consumptions. As physical wide PR array scales to an ultra-large size and temporal inputs becomes more complicated, this advantage would be essential to regulate the overall integration density in a system. Note that our scheme also can be combined with the preprocessing methods, which might result in a synergistic effect on the time processing capability. Further, our 3D stacked PR array enhances the versatility and flexibility of the wide RC system for a given task, because only the output layer needs to learn PR states response to newly incoming temporal information. Thanks to the satisfactory performances of PR states, there is almost no need to consider extra hardware designs, such as the lateral resistor connections for a multilayered structure and the parallel memristors scheme for low cycling variation.

With this point of view, we believe that our 3D wide PR array itself could allow to enable the seamless extraction of intricate 3D local features from biological moving cells and Lorenz attractors rather than utilizing additional preprocessing steps such as input masks (Figs. 4 and 5). Therefore, we believe that our approach makes the difference clear in terms of device, 3D architecture, and algorithmic operation, as well as their combinational functionalities.

Supplementary Note 2. Single and wide reservoir computing systems with WO_x memristor array

General reservoir computing

As shown in Supplementary Fig. 1a, typical reservoir computing (RC) system largely consists of two sections; a reservoir and output (or readout) layer.[8,9] A reservoir has stationary connectivity between the neurons regardless of time. Nevertheless, it can dynamically evolve with the time-dependent inputs ($u(t)$) while forming the reservoir states at a time t (i.e., $x(t)$) through the collective states of nonlinear neurons coupled with one another. In other words, $u(t)$ can be nonlinearly mapped into $x(t)$ in a new feature space via the reservoir, such that the resultant $x(t)$ become linearly separable. Hence, the $x(t)$ values are then readily analyzed by a single-layer neural network with a simple learning algorithm, which is performed at the output layer, the second section of the RC system. During the learning process, the weights W connected between the reservoir and output layer are updated and optimized toward certain desired values after which the RC system can conduct a given task. Therefore, the performance of the RC system is primarily determined by the computation capability in the reservoir, given

that the single-layer neural networks at the output layer are typically uncomplicated, which still remains challenging.

Single reservoir computing based on the WO_x memristors

To circumvent the above computational issue in the reservoir, WO_x-based memristors can be exploited for the realization of an efficient physical reservoir (Supplementary Fig. 1b), because they are able to implement the two essential properties for processing time-dependent inputs for the RC system; separation property and fading memory property.[8,9] The separation property means that different reservoir states are generated in response to different temporal inputs. The fading memory (i.e., short-term memory) property enables recurrent feedback between reservoir states, allowing a reservoir state at a certain time *t* to reflect its past history. Owing to the inherently nonlinear switching behavior and the spontaneous V_O diffusion, WO_x memristors can naturally implement a nonlinear dynamic reservoir with the two essential properties. For example, the internal states of the memristors depend on both programming voltage pulses at the prior and current timesteps due to the self-decaying property (short-term memory effect). Then, it is possible to readily represent the input timing information ($u(t)$) as current responses ($I(t)$ (i.e., $x(t)$). Note that the software-based reservoir interconnects the several hundreds or thousands of internal neurons to obtain the separation and fading memory effect. In contrast to this, a single WO_x memristor (i.e., a single physical node) with nonlinear dynamics can replace those complex nonlinearly coupled loops formed by multiple nodes in a software-based reservoir, enabling the simple implementation of the physical reservoir. In other words, the generation of reservoir states based on the WO_x memristors is readily enabled by applying temporal voltage signals, which could mitigate the complexity and calculations in the software-based reservoir. Note that the reservoir with more memristors can generally enrich the reservoir states and then further well-capture dynamics of the time information at different timescales. As a result, multiple memristors can be utilized for the larger capacity of the reservoir, improving the RC.

Wide reservoir computing based on the WO_x memristors

Generally, many dynamic systems or time-dependent information involve the multiple, nonlinear, and dimension-dependent variables. To achieve this, it must be ensured that the RC system extracts multiple local features from certain dynamic inputs.[10-12] This is challenging to accomplish in a single reservoir.[10-12] However, a wide RC that widens the topology of a single

RC can extract richer features from the temporal inputs by utilizing multiple reservoirs (Supplementary Fig. 1c). Because each reservoir (i.e., 3 layers) with its own characteristics can learn and address distinct local features, different time-varying inputs can be appropriately mapped into linearly separable $I_n(t)$ (n is the number of memristors) in a new feature space. Note that software-based approaches have demonstrated that the wide RC can have better computing performance than the single RC. Here, the wide RC system is implemented by vertically 3D integrating WO_x memristive crossbar arrays efficiently improving the physical reservoir capacity.

Supplementary Note 3. Benefits of our approach compared to prior applications

To date, several approaches such as backpropagation through time (BPTT) and recurrent neural networks (RNNs) have been already suggested to address time-series data.[13,14] However, this conventional approach necessitates the sequential unfolding and calculation of the neural network in accordance with timesteps (e.g., t-1, t, t+1), which results in a computationally intensive procedure (Supplementary Fig. 4). Furthermore, these methods are susceptible to the gradient vanishing (or exploding) issue. If the gradient is less or more than 1, the amount of weight updates becomes 0 or ∞ at a deeper timestep due to the accumulated multiplication of the gradient (Supplementary Fig. 4). This results in an ineffective learning process, which in turn restricts the size of the network and causes hardship for the long-term learning process.

In contrast to these approaches, reservoir computing (RC) can significantly reduce the learning costs since the learning process occurs only at the simple output layer by leaving the reservoir layer unchanged and fixed. Note that the output layer typically consists of single-layer network. Moreover, the gradient vanishing (or exploding) problem can be effectively avoided as it is unnecessary to multiply the gradients according to timesteps. Lastly, memristor-based RC can enhance the learning efficiency through their inherent nonlinearity and short-term memory capabilities by simplifying the reservoir operation rather than using software-based approaches. Therefore, memristor-based RC is recognized as a more effective method for processing diverse temporal information. From this point of view, previous demonstrations have shown that memristor-based RC can efficiently classify and predict spatiotemporal inputs as compared to conventional FFNs and digital computers.[15-19]

Nevertheless, the majority of these reported systems have been conventionally constructed around a single reservoir and employed 2D single-stack memristive frameworks.

Consequently, the ability of the single RC system to handle the multivariable dynamic inputs that are intrinsic to different types of time-series data can be compromised. This is mainly because the multiple local features are extracted and mapped into just one feature space.[10-12] Hence, RC performances would be naturally limited in terms of accuracy, reliability, and time (Figs. 4 and 5), which could be further deteriorated as the inputs include more local features. The objective of this study is to confront and resolve this matter.

To overcome this critical issue, we have physically increased the number of feature spaces by vertically stacked multiple memristive reservoirs using 3D-integrated array structure. As a result, we have demonstrated the following: Because each reservoir has its own feature space, the use of multiple reservoirs can individually capture distinct local traits of temporal information and nonlinearly transform them into multiple feature spaces, thus making RC systems more accurate, reliable, and rapid (Figs. 4 and 5). Furthermore, the 3D-integrated multilayered reservoirs can enable the parallel processing of different time-varying information unlike the sequential processing in 2D-based approaches. Therefore, we believe that our methodology offers evident advantages in both functionality and architecture, thereby facilitating the development of a streamlined and compact physical framework for RC.

Supplementary Note 4. 3D-integrated architecture

The type of 3D stacked memristive architectures can be typically classified as the horizontal type and the vertical type (Supplementary Fig. 5).[20,21] For the horizontal type of 3D stacked array, typical 2D crossbar arrays are layer-by-layer stacked on each other while forming a sidewall at each node edge, as shown in Supplementary Fig. 5a. This 3D array design, whose density ($4F^2/n$) increases with the number of stacked layers (n), is scalable laterally because memristors are vertically formed at each crosspoint. However, in this stacked method, each memristor should be combined with a selector (or transistor) to facilitate the selection and optimization of individual memristor in the 3D array. In addition, as the number of stacked layers increases, critical lithography and etching steps also increase despite the simple fabrication technology, leading to higher fabrication costs and lower vertical scalability. This array architecture inevitably forms undesired geometry such as rabbit ears and etched sidewalls at the junction edges, which could potentially lead to device failure and operational instability.

On the other hand, for the vertical type of 3D stacked array, as shown in Supplementary Fig. 5b, vertical pillars consisting of switching mediums are formed through multiple stacked plane electrodes, where each memristor is located between pillar and plane electrodes. In

general, this vertical type can streamline the lithography process in comparison to the horizontal one; consequently, the fabrication cost for the vertical direction may be somewhat reduced. However, as the stacked layer increases, the etching process becomes more challenging in order to generate deep holes with the intended ultrahigh aspect ratio. In addition, a stringent deposition technique is required to form switching layers and electrodes into the narrow and deep vertical holes. In this type, it is hard to integrate selectors (or transistors) on the individual memristor along the vertical pillars, rendering the architecture vulnerable to leakage current issues. Here, we summarize major features of the 3D stacked types in Supplementary Table 1.

Our proposed array architecture did not form the rabbit ears and etched sidewalls at the junction edges that could potentially lead to device failure and operational instability. As a result, the working device yield can be largely improved (Figs. 2b and 2c). In addition, the fabrication process for the layer-by-layer stack may be considerably less complex than the stacked methods of a conventional 3D memristor array. Based on the above major features, the advantages of our 3D-integrated multilayered PR architecture for the physical wide RC system over conventional stacked methods for 3D stacked memristor arrays are as follows:

Basically, our proposed stacked method is based on the horizontal type without rabbit ears and sidewalls due to the interface switching principle, in contrast to the conventional approach. In terms of the density and scalability, this 3D array design has density of $4F^2/n$ according to the number of stacked layers (n) and can be scalable laterally, which is identical to the horizontal type. However, our proposed array architecture did not form rabbit ears and sidewalls at the junction edges that could potentially lead to device failure and operational instability. As a result, the working device yield can be largely improved (Figs. 2b and 2c). In addition, the fabrication process for the layer-by-layer stack may be considerably less complex than the stacked methods of a conventional 3D memristor array. Our proposed stacked method eliminates the requirement for a selector or transistor in the WO_x memristor due to its self-rectifying switching properties. They become considerably simpler to stack or fabricate. As a result, the fabrication cost can be significantly lowered without the need of additional selectors (or transistors). With these reasons, we believe that our 3D architecture provides a practical 3D architecture for advanced reservoir systems in terms of the density, scalability, and cost.

Supplementary Note 5. Potential switching mechanism of WO_x memristor

Potential switching mechanism

Elemental compositions and chemical states of the fabricated Pt/WO_x/W memristor were investigated by using the depth-profiling XPS analysis with timed Ar^+ bombardment (Fig. 3a and Supplementary Fig. 15). After confirming the presence of the Pt, W, and O elements (Supplementary Fig. 15a), the WO_x switching layer was further examined according to the depth (Fig. 3a). Notably, the depth-dependent oxygen vacancies (V_O) distribution was obviously observed as shown in Fig. 3a. At the top WO_x interface, the binding energies of two peaks were located at 35.8 eV and 38.1 eV and coincided with the fully oxidized tungsten ions (W^{6+}),[22] indicating the depletion of V_O (i.e., x ~3 in WO_x) (top of Fig. 3a). By contrast, at the bottom WO_x interface, there were additional shoulders at lower binding energies with lower intensities corresponding to the W^{5+} (34.7 and 36.9 eV) and W^0 (31. 2 eV and 33. 4 eV),[22,23] indicating the enrichment of V_O (i.e., x<3 in WO_x) (bottom of Fig. 3a). Note that at the bottom W electrode, only metallic W^0 peaks were observed as shown in Supplementary Fig. 15b. Consequently, these findings suggest that our WO_x memristor includes the asymmetric profile of C_{V_O} within the WO_x layer (i.e., the increase in C_{V_O} in the top-bottom direction). Such V_O distribution within WO_x was in agreement with the dependency of the visual color on the WO_x thickness,[1] where the yellowish (x~3), bluish (2<x<3), and brownish (x~2) WO_x was observed as the thickness decreased (Supplementary Fig. 20). Moreover, the asymmetric profile of C_{V_O} within the WO_x can be further supported by the electrical characteristics. For example, I_{Sneak} was observed to increase with a decrease in WO_x thickness, implying that the Φ_B^2 becomes lower by the increased V_O (Supplementary Fig. 17b). Also, there is no noticeable change in *I*-*V* curves under a vacuum environment, revealing the possibility of more mobile V_O located near Φ_B^1 rather than Φ_B^2 (Supplementary Fig. 22). Additionally, the WO_x was observed to be amorphous phase with very small crystallites in the short-range order (Supplementary Fig. 23).

Due to the effect of the V_O on the interfacial barriers, it can be expected that the asymmetric interfacial barriers ($\Phi_B^1 < \Phi_B^2$) and the facile modulation at the Φ_B^1 are mainly attributable to the self-rectifying switching behaviors of the fabricated WO_x-based memristor. In this regard, as shown in Figs. 3b, 3c, and Supplementary Fig. 21, the Φ_B^1 and Φ_B^2 were quantitatively estimated by using the thermionic Schottky emission principle. Note that there is an assumption that the overall conduction is governed by the interfacial barrier near the cathode. Based on the fitting results in Fig. 3b (solid lines), the Φ_B^1 and Φ_B^2 for the ON and OFF states were individually evaluated as shown in Figs. 3c and Supplementary Fig. 21. With

these results, there are two noticeable points in the junction structure. One is that the junction consists of asymmetry interfacial barriers (i.e., $\Phi_B^1 < \Phi_B^2$). Another one is that the change range in Φ_B^1 is larger than Φ_B^2 during the switching (i.e., $\Delta\Phi_B^1 > \Delta\Phi_B^2$). This implies that the conductance states for I_{ON} and I_{OFF} are mainly determined by the change of the Φ_B^1. When the positive bias is applied to the top Pt electrode, the V_O can move downward to the bottom W electrode increasing C_{V_O}. Since the Fermi level of WO_x upshifts with increasing C_{V_O},[24] the Φ_B^1 can be largely lowered, resulting in the I_{ON}. In contrast, when the negative bias is applied to the top Pt electrode, all the behaviors occur inversely, switching to I_{OFF}. As a result, all these findings can explain the role of both interfaces upon switching; namely, the controllable Φ_B^1 for the switching and the robust Φ_B^2 for self-rectification. It should be noted that electron (de)trapping processes are not completely excluded and may partially contribute to the barrier modulation during the switching. Nevertheless, the electric field-driven V_O migration rather than the electron (de)trapping process can be regarded as the primary driving forces for the switching, because the opposite barrier modulation can occur if the electron (de)trapping at Φ_B^1 becomes dominant.[25,26]

Electron trapping and detrapping processes during the switching

Given the asymmetric V_O profile within the WO_x (Fig. 3a), it is reasonable to assume that the trap sites at the bottom WO_x/W interface is much lower than that at the top Pt/WO_x interface.[27] In this circumstance, electrons could be mainly captured and trapped near/at the top interface when a positive voltage is applied to the top electrode, which would lead to a decrease in currents by increasing the barrier height.[28] However, since the positive voltage can make the ON state (a higher current) in our WO_x memristor device system, it is reasonable to think that the dominant switching mechanism is mainly associated with the V_O migration near/at the bottom W/WO_x interface that can make the barrier modulation. Further, we have investigated and plotted temperature-dependent characteristics decaying time (t_o) via Arrhenius plot (Supplementary Fig. 24). As shown in Supplementary Fig. 24, the activation energy (E_A) for V_O migration was estimated to be ~0.20 eV, which is less than E_A of ~0.58 eV on the average in typical WO_x films ($2.8<x\leq3$).[29] This might be attributed to the combined effects between sputtering deposition parameters and electrodes, making the V_O more movable. Similarly, Park, S. et al. suggested that oxygen anions with E_A = ~0.21 eV could migrate across the whole TiO_x switching layer, leading to the switching behavior of the device.[30] From this result and above the discussion, we believe that electric field-driven V_O migration near/at Φ_B^1 can have a

substantial impact on the determination of the switching states. Nevertheless, because there is a possibility that the barrier modulation might be driven by the electron trapping, we did not completely exclude the effect of electron (de)trapping process on the switching.

Minimum currents at non-zero voltage

A minimum current at a certain voltage that results from a shift of the origin in the *I-V* switching curve may be caused by the transient formation of an internal electric field via the interplay between the trapped electrons and the transient gradient of V_O. For example, when a positive voltage ($+V_{input}$) is applied to the top Pt electrode, electrons could be trapped near/at the top interface. This trapping process could lead to a higher concentration of the negatively charged electrons at the top interface compared to the bottom interface. This transient accumulation of electrons at the top interface might generate the internal electric field (E_{int}) upward.[31,32] In addition to this, the $+V_{input}$ could concurrently migrate V_O from the top Pt electrode to the bottom W electrode, such that the positively charged V_O are relatively enriched near/at the bottom W electrode. The transient concentration gradient of V_O might also induce the E_{int} upward.[33] Hence, we suspect that the E_{int} driven by both two effects could offset the applied external electric field at a certain voltage point.

Asymmetric profile of oxygen vacancies

As shown in Supplementary Fig. 17, we examined a variety of Pt/WO_x/W memristors fabricated with WO_x deposition of varying thicknesses (Supplementary Fig. 17a and 17b). We found that the current responses in the negative voltage region were observed to be significantly suppressed as the thickness of WO_x increased from 20 nm to ≥40 nm. This implies the formation of a robust Schottky barrier at the Pt/WO_x interface as the thickness of WO_x increases, even if the whole WO_x layer is deposited under identical conditions. In other words, it indicates the concentration of oxygen vacancies (C_{V_O}) near the top WO_x interface decreases as the thickness of WO_x increases. This could be due to the possibility that oxygen gas or ions will occupy oxygen vacancies near or at the surface of WO_x as the processing time extends.[34,35] Furthermore, subsequent to the sputtering procedure, the heated WO_x surface may undergo partial oxidation as it cools in an air environment.[36] Similarly, Han et al. reported that N concentration in MnTe film sputtered under Ar/N_2 gas environment was observed to be ununiformly distributed according to the thickness despite the identical sputtering parameters.[37] In addition to sputter, it is known that other deposition tools such as PLD could

also induce the local vacancy concentration without the change in deposition parameters.[38,39] The O_2 ratio and substrate temperature during sputtering, which influence the distribution of V_O, are also critical fabrication parameters that impact both the self-rectifying and switching performances (Supplementary Figs. 17c and 17d). With these results, we believe that the degree of the asymmetric distribution of V_O may be influenced by the interaction between various sputtering parameters.

In addition, we confirmed the dependency on the used electrodes as shown in Supplementary Fig. 19. For example, the Pt/WO_x/Pt junction structure was observed to degrade the switching performances including small I_{ON}-I_{OFF} ratio, large operating voltage, low rectification ratio (Supplementary Fig. 19a). This result supports that the bottom Pt electrode cannot facilitate the adjustment of the interfacial barrier as compared to the bottom W electrode. Also, the W/WO_x/Pt memristor showed the reverse switching behavior but it failed to sustain the switching performances (Supplementary Figs. 17a and 19b), suggesting that the C_{V_O} is unevenly distributed in the direction of the bottom electrode. From these findings, it is important to determine the electrodes and their terminal positions (top or bottom) by considering the distribution of V_O in WO_x for desired switching behavior.

To further investigate the effect of different sputtering condition (i.e., substrate temp.) and bottom electrodes (Pt and W), we have performed additional depth-profiling XPS analyses of following junctions; Pt/WO_x/W and Pt/WO_x/W without substrate temperature (293 K). As shown in Figs. 3a and Supplementary Fig. 15, different bonding states of W 4f were observed at the top and bottom interfaces of the Pt/WO_x/W memristor, indicating the uneven profile of V_O. In contrast, the W 4f results for both interfaces of other memristor were almost similar, indicating the uniform profile of V_O (Supplementary Fig. 16) These observations suggest that the asymmetry of C_{V_O} within the WO_x might be attributed to the different substrate temperature.

Furthermore, the visual inspection of WO_x could support the dependency of V_O concentration on its thickness, as shown in Supplementary Fig. 20. As shown in Supplementary Fig. 20, the top surface of WO_x/W on SiO_2/Si substrate was appeared in brown, blue, and yellow as the thickness of WO_x increased (Supplementary Fig. 20). These results correspond to WO_2, WO_x ($2<x<3$), and WO_3, respectively.[1] With these results, we do believe that the Pt/WO_x/W memristor operates based on the asymmetric distribution of oxygen vacancies in WO_x between Pt and W electrodes.

Supplementary Note 6. Analyses and relevant discussions of wide physical reservoir computing based on multiple 3D stacked WO_x physical reservoirs

Requirements of the memristor for wide physical reservoir computing

There are several requirements in the memristor to realize a wide physical RC such as separation and fading (short-term) memory properties, nonlinearity, and high dimensionality in the memristor.[8,9] The two essential separation and fading memory properties were already discussed in Supplementary Note 2. The nonlinear responses of a physical reservoir are also important because a reservoir should be able to nonlinearly map from incoming inputs. In other words, the nonlinearity of the reservoir facilitates the transformation from the inputs with similar features to the outputs in a certain range. Then, it allows the reservoir outputs to be readily analyzed and learned at the output layer with a simple algorithm. In addition to this, it can extract certain nonlinear dependencies between incoming inputs to predict their future behaviors. In the fabricated 3D stacked WO_x array, nonlinear current outputs were generated in response to voltage inputs (Fig. 2a), which can aid in nonlinearly mapping the temporal inputs into a new feature space. High dimensionality or stacked degree in the array level is another factor for determining the performance of the physical RC (Supplementary Note 2). It can either improve the separation property or represent various time dependencies among inputs. Also, the richness of reservoir states can be enhanced with the dimensionality. Therefore, our 3D stacked WO_x array architecture can be beneficial to implement the wide physical RC.

Furthermore, there are also tunable parameters to set up a physical reservoir, including the number of memristors and decaying time. The effective reservoir size for the capacity can be determined by the number of memristors that increases the number of reservoir nodes to enrich reservoir states. A larger reservoir capacity can bring better computing capability. Nonetheless, it will inevitably consume more energy and require a longer time during reservoir operations. Therefore, the number of memristors may be appropriately adjusted according to the types of a given task. In our 3D stacked WO_x array, however, the number of memristors required for the same task can be reduced compared to a single reservoir, and classification accuracy can be improved. (60% fewer memristors are required for dimensional classification of biological cell positions than for a single reservoir, as depicted in Figure 4). Moreover, the decaying time of memristors can alter the timescale in dynamics of a physical reservoir. For example, a shorter decaying time leads to a faster reservoir, whereas a larger decaying time

brings about a slower reservoir. It can be expected that an optimal decaying time might depend on timescales of a given inputs. Because the fabricated WO_x-based memristors exhibited a tunable decaying time with different programming voltage schemes (Figs. 2g and 4a), it can be tailored for a specific decaying time. As a result, there is a possibility that our hardware approach could implement various reservoir configurations to address different types of time-series data.

Convergence speed for wide physical reservoir computing

Further, the convergence (optimization) speed in the RC systems can be determined by the reservoir capacity. This is because a large reservoir capacity can efficiently enable feature extraction with a smaller number of memristors in each reservoir as shown in Fig. 4i. Because the number of the weights to be trained at the output layer decreases with the number of memristors, the multiple reservoirs system could accelerate the convergence speed with 60 % fewer memristors as compared to the single reservoir system (Fig. 4i). Similarly, the effect of the reservoir size on the convergence speed has been previously investigated.[40] Note that the prediction accuracy in the wide RC increases despite the use of fewer memristors (Fig. 4i), verifying that stacked reservoir layers can effectively improve the reservoir capacity rather than just expanding the size of the single reservoir (i.e., the number of memristors). Therefore, the wide RC can make the learning process faster based on the enhanced reservoir capacity.

Efficiency of wide physical reservoir computing compared to conventional approaches

As shown in Supplementary Fig. 33, we have performed the prediction of Lorenz attractor via one of the conventional standard deep learning models, 100-1000-1000-1 multilayered perceptron (MLP) consisting of an input, two hidden, and an output layer (Supplementary Fig. 33). Here, as a good comparator, we have only considered the prediction of Lorentz attractor data. This is because this task has significantly more timesteps than biological cell images, which can show the stark difference in efficiency between the two platforms. In this simulation, similar to the configuration of wide physical reservoir computing (RC), we employed three MLPs for predicting *x*, *y*, and *z* components of Lorenz attractor (Supplementary Fig. 33a). Moreover, because the input at a certain timestep is not stored and is independent of each other in typical MLPs, inputs at the current timestep (*t*) and the previous timesteps (*t*-1, *t*-2 …, *t*-99) were simultaneously fed to the networks during learning processes (Supplementary Fig. 33b), where loss function, learning rule, learning epoch, and batch size

are mean square error, RMSprop (learning rate = 0.01 and exponential decay=0.9), 200, and 2, respectively. The activation function of the hidden and output layers was set to rectified linear unit (ReLU) and linear function with bias, respectively. After learning processes until 1400 timesteps, as shown in Supplementary Figs. 33c and 33d, predicted behavior largely deviates the actual behavior. The average normalized mean squared errors (NMSEs) were estimated to be ~9.46×10^{-2}. This result is >300 times worse than that of the wide physical RC (~2.62×10^{-4}), although the MLP approach has the much larger number of learning parameters (i.e., 1,103,001 × 3 = 3,309,003) than that of the wide physical RC (100 × 3 = 300). This might be attributed to the insufficient capability to capture certain nonlinear dependencies between sequential incoming inputs. Similarly, for the prediction of chaotic Mackey-glass equation, single physical RC has already shown better prediction results than deep learning models such as MLP and long short-time memory (LSTM) with much fewer learning parameters.[17] Consequently, these results support that physical RC can efficiently process time-series data at lower learning cost.

Energy consumption for wide physical reservoir computing and conventional computing

Although there is rarely a report or estimation regarding the energy consumption for bio cell position classification and Lorentz attractor prediction using conventional digital systems featuring von Neumann architecture, here we have compared the energy cost required to perform Lorentz attractor prediction in a wide RC system based on the conventional software reservoirs and the 3D-integrated multilayered PR array based on a few assumptions for simple calculation. Basically, Lorentz attractor data have significantly more timesteps than biological cell images, leading to the stark difference in energy consumption between the two platforms, which can be a good comparative factor.

To estimate the energy consumed by the conventional computing system, we have utilized the Intel 11th Generation Core i7-1165G7 @ 2.80GHz and a software-based real-time power monitoring program based on the Running Average Power Limit (RAPL) interface.[41,42] This program can capture real-time energy consumption of the cores, arithmetic logic unit (ALU), floating point unit (FLU), integrated graphics, and DRAM memory controller, and others. The idle energy (E_{idle}) per second that keeps the computer just on without any running programs was estimated to be ~2.63 Js^{-1} on average. Because the operating energy (E_{op}) that runs a certain program in the computer includes both the program running energy (E_{run}) and the idle energy, the difference between E_{op} and E_{idle} can become E_{run} (i.e., $E_{run} = E_{op} - E_{idle}$). To

obtain E_{run} for the wide RC, each node of software-based reservoirs was set to follow the typical formula:

$$x_t = (1-\alpha)x_{t-1} \times \alpha\tanh(W_{in} \times u_{t-1}) + W_{res} \times x_{t-1}, \quad (4)$$

where x_t, α, x_{t-1}, W_{in}, u_{t-1}, and W_{res} are reservoir states at current timesteps, leak factor, reservoir states at prior timestep, input weights between input and reservoir, input data at prior timestep, and reservoir weights within the reservoir, respectively. For the unbiased comparison, other parameters of the reservoirs such as the number of reservoirs (3), the number of nodes at each reservoir (100), and the operating scheme were set to almost match with the case of physical wide RC (Method section of manuscript). As a result, the E_{op} required for the software-based multiple reservoirs was observed to be 53.15 J for 10.50 s, such that the E_{run} was estimated to be ~25.52 J (~53.15 J (E_{op}) – 27.63 J (E_{idle} × time spent)). Here, we can approximate the energy cost of the reservoir operation for each node and input (E_{node}), namely, $E_{node} = E_{run}$ / (total node number × input length) = ~25.52 J/(300 × 2788) = 3.05×10^{-5} J.

For the 3D stacked array, the energy consumed at each reservoir node (i.e., memristor) and each input (E_{mem}) can be estimated by

$$E_{mem} = V_{\mathrm{INPUT}} \times I \times W, \quad (5)$$

where V_{INPUT}, I, and W are input voltage, current response, and pulse width, respectively. When the V_{INPUT} and I were set to be 1.8 V and ~10^{-5} A to represent their maximum possible values, the E_{mem} was estimated to be from ~3.60×10^{-12} J to 1.80×10^{-8} with respect to the W range from 200 ns to 1 ms. Based on these estimations, we can say that the reservoirs of the 3D-integrated multilayer PR array could decrease the consumed energy by up to ~8.47×10^{6} times compared to that of the conventional computing system. Therefore, we believe that this result supports the aim of implementing a physical wide RC system based on the 3D stacked PR array to efficiently process multiple dynamic time-series information.

Suggestion of circuit-level implementation of the physical wide reservoir computing

Because the 3D stacked array itself cannot operate without the help of peripheral circuits, it is important to discuss how to entirely implement the wide RC system at the circuit level. Hence, we believe that this suggestion and relevant discussion are obviously valuable for improving the quality of our study, offering a practical approach to the applicability of the proposed 3D-integrated multilayered PR array. Nevertheless, we would like to explain and justify why the simulation results based on the 3D stacked PR array still stand effective for a

proof of principle demonstration. RC can significantly reduce the learning costs since the learning process occurs only at the simple output layer by leaving the reservoir layer unchanged and fixed. Note that the output layer typically consists of single-layer network. In other words, the implementation of efficient reservoir layer is most important to realize RC systems because conventional digital computers can fully afford training the single-layer network. With this point of view, many studies on physical RC systems have focused on the implementation of reservoir layer, especially based on memristors. This is mainly because memristors can enhance RC efficiency through their inherent nonlinearity and short-term memory capabilities by simplifying the reservoir operation rather than using software-based approaches. Hence, by simulating and modeling RC with PR states of the memrsitive PRs, the physical RC systems have been demonstrated with an emphasis on the applicability of the proposed PRs.[30,43,44] In this sense, we believe that the simulation results, based on the fabricated 3D stacked wide PR array (Figs. 4a, 4g, Supplementary Fig. 27-30), can sufficiently highlight the importance and implications of our study, despite the absence of full hardware implementation at the circuit level. Therefore, here, we would like to discuss how to implement the wide RC system based on previous literature (single RC by memristor array) and our following suggested circuit diagram.

As in Ref 45, we have demonstrated a memristive physical single RC system at the circuit level, where Ag-based memristors and 1T1M array were utilized for the PR and output layer, respectively. Briefly, when sequential voltage pulses were applied to the PR, the current responses were transferred to a customized MATLAB code that communicated with a micro-controller unit (MCU) via serial ports. Since the digital input/output (I/O) ports of MCU were wired with digital-to-analog converters (DACs), analog-to-digital converters (ADCs), and transimpedance amplifiers (TIAs) on the printed circuit board (PCB), the output layer could perform the training and inference processes with respect to the time-dependent voltage pulses. Based on our previous approach, we think that the similar peripheral circuitries could be applied to the suggested physical wide RC, as illustrated and described in Supplementary Fig. 34.

As shown in the left of Supplementary Fig. 34, from this hardware design, time-dependent voltage inputs can be applied to 3D stacked PR array consisting of three PRs, which can result in the corresponding current responses (i.e., reservoir states). Then, these current signals can be converted to voltage signals via transimpedance amplifiers (TIAs), which can be also amplified via output amplifier if necessary. Then, the resultant signals are delivered

into the PC/MCU block. The PC is for user interface that can control data and PCB boards through a customized code program. The MCU communicates with the PC, controls the boards via I/O ports, and generates/reads signals via DACs and ADCs. As shown in the right of Supplementary Fig. 34, 1T1M could be utilized for the output layer that can learn and infer the reservoir states from the 3D stacked PR array. For the column blocks, multi-channel DAC can apply voltages into the gates of transistors and the top electrodes of the memristors at the output layer. Also, TIAs, multiplexers (MUXs), and ADC are responsible for the sensing the current. For the row blocks, MUXs and latches interfacing with DACs can supply voltages to the bottom electrodes of the memristors at the output layer. All column and row blocks can incorporate a MCU interfaced with a PC via serial-port communication. Based on the suggested circuit diagrams, we think that the wide RC system using a 3D-integrated multilayered PR array could operate at a circuit level.

Because it is necessary to design and develop several essential circuits in-depth, for example, the parallel signaling and its conversion schemes, operation of the output layer, and fabrication of 1T1R array, we think that the direct implementation of the wide physical RC system at the circuit level is beyond the current study and subject. Nevertheless, we believe that this discussion provides a circuit framework for the wide physical RC system using the 3D-integrated multilayered PR array.

Supplementary References

1 Weil, M. & Schubert, W.-D. The beautiful colours of tungsten oxides. *International tungsten Industry Association* (ITIA) **4**, 1-12 (2013).

2 Wang, M. *et al.* Electric-field-controlled phase transformation in WO3 thin films through hydrogen evolution. *Adv. Mater.* **29**, 1703628 (2017).

3 Cannavale, A. *et al.* Highly efficient smart photovoltachromic devices with tailored electrolyte composition. *Energy Environ. Sci.* **4**, 2567-2574 (2011).

4 Johnson, K. L. & Johnson, K. L. *Contact mechanics*. (Cambridge university press, 1987).

5 Pezzoli, A. *et al.* Thermal annealing and exposure to divertor-like deuterium plasma of tailored tungsten oxide coatings. *J. Nucl. Mater.* **463**, 1041-1044 (2015).

6 Liu, K. *et al.* Multilayer Reservoir Computing Based on Ferroelectric α-In2Se3 for Hierarchical Information Processing. *Adv. Mater.* **34**, 2108826 (2022).

7 Sun, W. *et al.* 3D Reservoir Computing with High Area Efficiency (5.12 TOPS/mm2) Implemented by 3D Dynamic Memristor Array for Temporal Signal Processing. in *2022 IEEE Symposium on VLSI Technology and Circuits (VLSI Technology and Circuits).* 222-223 (IEEE).

8 Nakajima, K. Physical reservoir computing—an introductory perspective. *Jpn J. Appl. Phys.* **59**, 060501 (2020).

9 Tanaka, G. *et al.* Recent advances in physical reservoir computing: A review. *Neural Netw.* **115**, 100-123 (2019).

10 Carmichael, Z., Syed, H., Burtner, S. & Kudithipudi, D. Mod-deepesn: modular deep echo state network. preprint at https://arxiv.org/abs/1808.00523 (2018).

11 Han, M. & Mu, D. Multi-reservoir Echo State Network with Sparse Bayesian Learning. in *Advances in Neural Networks-ISNN 2010: 7th International Symposium on Neural Networks, ISNN 2010, Shanghai, China, June 6-9, 2010, Proceedings, Part I 7.* 450-456 (Springer, 2010).

12 Meftah, B., Lézoray, O. & Benyettou, A. Novel approach using echo state networks for microscopic cellular image segmentation. *Cogn. Comput.* **8**, 237-245 (2016).

13 Werbos, P. J. Backpropagation through time: what it does and how to do it. *Proceedings of the IEEE* **78**, 1550-1560 (1990).

14 Medsker, L. & Jain, L. C. *Recurrent neural networks: design and applications*. 1st ed. (CRC press, 1999).

15 Kulkarni, M. S. & Teuscher, C. Memristor-based Reservoir Computing in *2012 IEEE/ACM International Symposium on Nanoscale Architectures.* (NANOARCH) 226-232 (2012).

16 Du, C. *et al.* Reservoir computing using dynamic memristors for temporal information processing. *Nat. Commun.* **8**, 2204 (2017).

17 Moon, J. *et al.* Temporal data classification and forecasting using a memristor-based reservoir computing system. *Nat. Electron.* **2**, 480-487 (2019).

18 Milano, G. *et al.* In materia reservoir computing with a fully memristive architecture based on self-organizing nanowire networks. *Nat. Mater.* **21**, 195-202 (2022).

19 Zhong, Y. *et al.* A memristor-based analogue reservoir computing system for real-time and power-efficient signal processing. *Nat. Electron.* **5**, 672-681 (2022).

20 Yoon, K. J., Kim, Y. & Hwang, C. S. What will come after V-NAND—vertical resistive switching memory? *Adv. Electron. Mater.* **5**, 1800914 (2019).

21 Li, Y. & Ang, K.-W. Hardware implementation of neuromorphic computing using large-scale memristor crossbar arrays. *Adv. Intell. Syst.* **3**, 2000137 (2021).

22 Wang, G. *et al.* Hydrogen-treated WO_3 nanoflakes show enhanced photostability. *Energy Environ. Sci.* **5**, 6180-6187 (2012).
23 Katoh, M. & Takeda, Y. Chemical state analysis of tungsten and tungsten oxides using an electron probe microanalyzer. *Jpn J. Appl. Phys.* **43**, 7292 (2004).
24 Lambert-Mauriat, C., Oison, V., Saadi, L. & Aguir, K. Ab initio study of oxygen point defects on tungsten trioxide surface. *Surf. Sci.* **606**, 40-45 (2012).
25 Lee, J. S., Lee, S. & Noh, T. W. Resistive switching phenomena: A review of statistical physics approaches. *Appl. Phys. Rev.* **2**, 031303 (2015).
26 Seong, D.-j., Jo, M., Lee, D. & Hwang, H. HPHA effect on reversible resistive switching of Pt/Nb-doped $SrTiO_3$ Schottky junction for nonvolatile memory application. *Electrochem. Solid-state Lett.* **10**, H168 (2007).
27 Wu, L., Liu, H., Lin, J. & Wang, S. Volatile and Nonvolatile Memory Operations Implemented in a Pt/HfO_2/Ti Memristor. *IEEE Trans. Electron Devices* **68**, 1622-1626 (2021).
28 Nicholls, J. R., Dimitrijev, S., Tanner, P. & Han, J. The role of near-interface traps in modulating the barrier height of SiC Schottky diodes. *IEEE Trans. Electron Devices* **66**, 1675-1680 (2019).
29 Le, H. M., Vu, N. H. & Phan, B.-T. Migrations of oxygen vacancy in tungsten oxide (WO_3): A density functional theory study. *Comput. Mater. Sci.* **90**, 171-176 (2014).
30 Park, S.-O., Jeong, H., Park, J., Bae, J. & Choi, S. Experimental demonstration of highly reliable dynamic memristor for artificial neuron and neuromorphic computing. *Nat. Commun.* **13**, 2888 (2022).
31 Wang, G. *et al.* Three-dimensional networked nanoporous Ta_2O_{5-x} memory system for ultrahigh density storage. *Nano Lett.* **15**, 6009-6014 (2015).
32 Choi, S. *et al.* A self-rectifying TaO y/nanoporous TaO x memristor synaptic array for learning and energy-efficient neuromorphic systems. *NPG Asia Materials* **10**, 1097-1106 (2018).
33 Sun, B. *et al.* Non–zero-crossing current-voltage hysteresis behavior in memristive system. *Mater. Today Adv.* **6**, 100056 (2020).
34 von Wenckstern, H. *et al.* Schottky contacts to In_2O_3. *APL Mater.* **2** (2014).
35 Veal, B. W. *et al.* Interfacial control of oxygen vacancy doping and electrical conduction in thin film oxide heterostructures. *Nat. Commun.* **7**, 11892 (2016).
36 Zhu, J. *et al.* Probing vacancy behavior across complex oxide heterointerfaces. *Sci. Adv.* **5**, eaau8467 (2019).
37 Han, Y. *et al.* Combinatorial nitrogen gradients in sputtered thin films. *ACS Comb. Sci.* **20**, 436-442 (2018).
38 Schneider, C. *et al.* The origin of oxygen in oxide thin films: Role of the substrate. *Appl. Phys. Lett.* **97** (2010).
39 Chen, Y. *et al.* Metallic and insulating interfaces of amorphous SrTiO3-based oxide heterostructures. *Nano Lett.* **11**, 3774-3778 (2011).
40 Porte, X., Andreoli, L., Jacquot, M., Larger, L. & Brunner, D. Reservoir-Size Dependent Learning in Analogue Neural Networks. In *Artificial Neural Networks and Machine Learning – ICANN 2019: Workshop and Special Sessions. ICANN* **11731**, 184-192 (2019).
41 Khan, K. N., Hirki, M., Niemi, T., Nurminen, J. K. & Ou, Z. RAPL in Action: Experiences in Using RAPL for Power measurements. *ACM Transactions on Modeling and Performance Evaluation of Computing Systems (TOMPECS)* **3**, 1-26 (2018).

42 Prieto, B., Escobar, J. J., Gómez-López, J. C., Díaz, A. F. & Lampert, T. Energy efficiency of personal computers: a comparative analysis. *Sustainability* **14**, 12829 (2022).
43 Jang, Y. H. *et al.* Time-varying data processing with nonvolatile memristor-based temporal kernel. *Nat. Commun.* **12**, 5727 (2021).
44 Sun, L. *et al.* In-sensor reservoir computing for language learning via two-dimensional memristors. *Sci. Adv.* **7**, eabg1455 (2021).
45 Midya, R. *et al.* Reservoir computing using diffusive memristors. *Adv. Intell. Syst.* **1**, 1900084 (2019).
